# Supplementary material for: Rebound Inverts the Staphylococcus aureus Bacteremia Prevention Effect of Antibiotic Based Decontamination Interventions in ICU Cohorts with Prolonged Length of Stay
Source: Antibiotics (Basel). 2024 Mar 29;13(4):316. doi: 10.3390/antibiotics13040316 (PMC11047347; doi:10.3390/antibiotics13040316)

Additional file contents:

Table S1: Observational studies (Benchmark groups) 2 - 6
Table S2: Studies of non-decontamination-based methods of VAP prevention 7- 10
Table S3: Studies of topical antiseptic based methods of VAP prevention 10-11
Table S4: Studies of antibiotic based methods of VAP prevention 12-15
Table S5. Meta-regression models of prevention effect size versus LOS 16
Table S6. Meta-regression models of *S aureus* infection incidence versus LOS 17
References S1 – S294 18 – 33
Figure S1: S aureus VAP prevention effect sizes; Non decontamination interventions 34
Figure S2: S aureus VAP prevention effect sizes; Decontamination interventions 35
Figure S3: S aureus BSI prevention effect sizes; All studies 36
Figure S4: Meta-regression S aureus VAP prevention effect sizes, Non decontamination studies 37
Figure S5: Meta-regression S aureus BSI prevention effect sizes, Non decontamination studies 37
Figure S6: Meta-regression S aureus VAP prevention effect sizes, Anti-septic studies 38
Figure S7: Meta-regression S aureus BSI prevention effect sizes, Anti-septic studies 38
Figure S8: Meta-regression S aureus VAP prevention effect sizes, Antibiotic studies 39
Figure S9: Meta-regression S aureus BSI prevention effect sizes, Antibiotic studies 39
Figure S10 & Figure S11: S aureus VAP incidence among observational cohorts & NCC groups 40
Figure S12: GSEM of postulated model 41

Table S1. Observational studies (Benchmark groups).

Author	Year	Ref	Notes	LOS	Patients (n)	V- S aureus (n)	V- S aureus %	Patients (n)	B- S aureus (n)	B- S aureus %
**A'court**	1993	1	Cohort	12	150	4	2.7	150	5	3.3
**Alvarez-Lerma**	1996	2	Cohort	7	6494	102	1.6			
**Apostolopoulou**	2003	3	Cohort	16	175	9	5.1			
**Arroliga_all**	2012	4	combined	8	2781	20	0.7			
**Arumugam**	2018	5	All	7	332	16	4.8			
**Baldesi**	2017	6		7	.	.	.	246459	1490	0.6
**Bekaert**	2011	7	Cohort	8	4479	133	3.0			
**Bercault_all**	2005	8	Cohort	10	236	4	1.7			
**Blot >74**	2014	9		8	516	17	3.3			
**Blot 65_74**	2014	9		8	549	22	4.0			
**Blot 45_64**	2014	9		8	670	27	4.0			
**Bochicchio**	2004	10	Cohort	13	678	50	7.4			
**Bonten'94**	1994	11	Cohort	25	64	3	4.7			
**Boots'06_All**	2006	12	All	12	381	28	7.3			
**Boots**	2008	13	Cohort	13	412	32	7.8			
**Bornstain**	2004	14	Cohort	12	747	17	2.3			
**Braun**	1986	15	Cohort	7	66	6	9.1			
**Bregeon**	1997	16	VAP	11	660	34	5.2			
**Cade**	1993	17	Cohort	16	98	13	13.3	98	4	4.1
**Cavalcanti**	2006	18	Cohort	10	190	18	9.5			
**Cenderero**	1999	19	Cohort	7	123	9	7.3			
**Chaari**	2015	20	Cohort	8	175	6	3.4			
**Chastre**	1998	21	Cohort	14	243	44	18.1			
**Chevret**	1993	22	Cohort	5	255	23	9.0			
**Combes**	2000	23	CTSS	18	104	4	3.8			
**Cook**	2010	24	Trauma	8	511	15	2.9			
**Cook**	2010	24	Non-trauma	13	2080	14	0.7			
**Craven-medical**	1988	25	Cohort	6	277	9	3.2	526	4	0.8
**Craven-surgical**	1988	25	Cohort	6	521	12	2.3	799	10	1.3
**Daschner**	1988	26	Cohort	6	116	13	11.2			
**Delclaux**	1997	27	ARDS	25	30	5	16.7			
**Delle Rose**	2016	28	Cohort	5	1647	32	1.9			
**Edgeworth**	1999	29		5				9272	36	0.4
**El-Masri**	2004	30		11	.	.	.	361	24	6.6
**Ensminger**	2006	31	Cohort	7	92	6	6.5			
**Ertugrul**	2006	32		10	100	12	12.0	100	9	9.0
**Esnault**	2017	33	Cohort	10	175	33	18.9			
**Evans**	2010	34	Cohort	8	416	40	9.6			

Table S1 (continued): Observational studies (Benchmark groups).

Author	Year	Ref	Notes	LOS	Patients (n)	V- S aureus (n)	V- S aureus %	Patients (n)	B- S aureus (n)	B- S aureus %
**Ewig**	1999	35	Cohort	10	48	5	10.4			
**Fabian_all**	1993	36	All	11	278	32	11.5			
**Fagon'89**	1989	37	Cohort	13	567	17	3.0			
**Garcı´a-Garmendia**	2001	38		5	.	.	.	2640	39	1.5
**Garrouste-Orgeas**	1997	39	Cohort	11	86	13	15.1			
**Garrouste-Orgeas**	2006	40		11		.	.	3247	46	1.4
**George**	1998	41	Cohort	8	223	8	3.6			
**Georges**	2000	42	Cohort	20	135	11	8.1			
**Giard**	2008	43	Cohort	9	7236	193	2.7			
**Gouel-Cheron**	2022	44		4				143589	185	0.1
**Gruson-97-98**	2000	45	Cohort	13	1029	54	5.2			
**Gruson-95-96**	2000	45	Cohort	13	1004	67	6.7			
**Gruson-99-01**	2003	46	Cohort	13	823	26	3.2			
**Guérin**	1997	47	Cohort	19	260	3	1.2			
**Guimaraes**	2006	48	Cohort	8	278	7	2.5			
**Guimaraes**	2006	48	Cohort	8	278	7	2.5			
**Gursel**	2010	49	Cohort	10	92	13	14.1			
**Heyland**	1999	50	Cohort	7	1014	64	6.3			
**Holzapfel_93**	1993	51	All	10	300	8	2.7	300	6	2.0
**Hortal**	2009	52	Cohort	10	231	38	16.5			
**Hugonnet**	2007	53	Cohort	6	936	55	5.9			
**Hyllienmark**	2007	54		4	221	2	0.9			
**Ibáñez**	2000	55	Cohort	8	30	3	10.0			
**Ibrahim'00**	2000	56	Cohort	5	1882	143	7.6			
**Ibrahim'00**	2000	57		10.5	.	.	.	4913	94	1.9
**Jacobs**	1990	58	Cohort	15	24	2	8.3			
**Jaillette**	2011	59	Cohort	15	439	22	5.0			
**Jaimes**	2007	60	Cohort	11	270	10	3.7			
**Jimenez**	1989	61	Cohort	10	77	2	2.6			
**Kallel**	2005	62	case control	15	241	15	6.2			
**Kallel**	2020	63	case control	26	2353			2353	57	2.4
**Kanafani**	2003	64	Cohort	17	70	3	4.3			
**Kantorova**	2004	65	All	9	287	5	1.7			
**Karin**	2020	66	Cohort	12	92	1	1.1			

Table S1 (continued): Observational studies (Benchmark groups).

Author	Year	Ref	Notes	LOS	Patients (n)	V- S aureus (n)	V- S aureus %	Patients (n)	B- S aureus (n)	B- S aureus %
**Kasuya**	2011	67	stroke	14	111	19	17.1			
**Kirschenbaum**	2002	68	kinetic bed	21	37	1	2.7			
**Ko**	2013	69		23	1453		.	1453	16	1.1
**Kollef' 93**	1993	70	Cohort	7	277	9	3.2			
**Kollef '95**	1995	71	All	7	300	22	7.3			
**Kollef '95**	1995	72	Cohort	16	314	17	5.4			
**Kollef '97**	1997	73	Cohort	8	521	25	4.8			
**Kollef '97**	1997	74	HH	5	147	7	4.8			
**Kollef**	2014	75	Cohort	11	1873	65	3.5			
**Kolpa**	2018	76		19	1270	16	1.3	1847	10	0.5
**Koss– N**	2001	77	Cohort	14	87	3	3.4			
**Koss– P**	2001	77	Cohort	11	66	4	6.1			
**Kunac**	2014	78	Cohort	6	716	62	8.7	206	6	2.9
**Lambert**	2011	79		5	.	.	.	119699	462	0.39
**Laupland**	2002	80		4.9	.	.	.	1017	18	1.8
**Laupland**	2004	81		5	.	.	.	4473	45	1
**Leblebicioglu**	2013	82	intervention	8	3864	51	1.3			
**Leblebicioglu**	2013	82	baseline	6	448	14	3.1			
**Lepelletier**	2010	83	Cohort	18	161	34	21.1			
**Lowy**	1987	84	Cohort		78	5	6.4			
**Luna**	2003	85		8	427	19	4.4			
**Luyt**	2005	86	Cohort	30	290	12	4.1			
**Magnason**	2008	87	Cohort	8	280	1	0.4	280	1	0.4
**Magret**	2010	88	Cohort	12	2436	116	4.8			
**Mahul**	1992	89	Cohort	22	145	10	6.9			
**Makris**	2011	90	Cohort	22	152	3	2.0			
**Markowicz**	2000	91	Cohort	10	744	74	9.9			
**Markowicz**	2000	91	ARDS	20	134	19	14.2			
**Massart**	2021	92		6	.	.	.	2464	13	0.53
**Meduri**	1998	93	ARDS	24	94	10	10.6			
**Memish**	2000	94	Cohort	11	202	16	7.9			
**Michel**	2005	95	Cohort	11	299	12	4.0			
**Moine**	2002	96	Cohort	14	764	19	2.5			
**Myny**	2005	**97**	Cohort	4	385	27	7.0			
**Nguile-Makao**	2010	98	Cohort	7	2873	89	3.1			
**Nielsen**	1992	99	Cohort	5	242	5	2.1			

Table S1 (continued): Observational studies (Benchmark groups).

Author	Year	Ref	Notes	LOS	Patients (n)	V- S aureus (n)	V- S aureus %	Patients (n)	B- S aureus (n)	B- S aureus %
**Noor**	2005	100	Cohort	7	250	11	4.4			
**Nseir**	2005	101	case control	10	1241	15	1.2			
**Osmon**	2003	102		8.3	893	.	.	893	29	3.2
**Outcomerea**	2019	103	Cohort	8	7735	258	3.3			
**Papazian**	1996	104	Cohort	10	586	20	3.4			
**Pawar**	2003	105		4	952	4	0.4			
**Plurad**	2013	106	Cohort	12	94	8	8.5			
**Pouwels**	2018	107		7	.	.	.	3159	69	2.2
**Prowle**	2011	108		6	.	.	.	6339	88	1.4
**Ramirez**	2016	109		13	440	8	1.8			
**Rello'91**	1991	110	Cohort	8	264	15	5.7			
**Rello'94**	1994	111		7	.	.	.	1650	16	0.97
**Rello'02**	2002	112	Cohort	8	9080	143	1.6			
**Rello'03**	2003	113	Cohort	20	99	2	2.0			
**Resende**	2013	114	Cohort	22	126	5	4.0			
**Reusser**	1989	115	Cohort	7	40	6	15.0	40	1	2.5
**Rezai**	2017	116	Cohort	10	562	24	4.3			
**Rincón-Ferrari**	2004	117	Cohort	10	310	27	8.7			
**Rodrigues**	2009	118	Cohort	10	233	11	4.7			
**Rodriguez**	1991	119	Cohort	14	294	37	12.6			
**Rosenthal_A**	2006	120		6	8867	91	1.0			
**Rosenthal_B**	2006	120		10	1029	19	1.8			
**Rosenthal_C**	2006	120		7	2172	25	1.2			
**Rosenthal_D**	2006	120		5	3413	1	0.0			
**Rosenthal_F**	2006	120		7	410	1	0.2			
**Rosenthal_E**	2006	120		6	1514	10	0.7			
**Rosenthal_G**	2006	120		5	1359	21	1.5			
**Rosenthal_H**	2006	120		12	2305	118	5.1			
**Rosenthal**	2012	121	baseline	7	3889	21	0.5			
**Rosenthal**	2012	121	intervention	6	51618	319	0.6			
**Ruiz-Santana**	1987	122	Cohort	7	1005	12	1.2			
**Salata**	1987	123	Cohort	11	51	2	3.9			
**Shahin**	2013	124	Cohort	10	267	6	2.2			
**Sofianou**	2000	125	Cohort	30	198	13	6.6			
**Stéphan**	2006	126	Cohort	16	175	43	24.6			
**Stolcin**	2020	127	Cohort	6	930	47	5.1	3388	11	0.32
**Sutherland**	1995	128	ARDS	20	105	7	6.7			

Table S1 (continued): Observational studies (Benchmark groups).

Author	Year	Ref	Notes	LOS	Patients (n)	V- S aureus (n)	V- S aureus %	Patients (n)	B- S aureus (n)	B- S aureus %
**Tan**	2016	129		16	264	10	3.8			
**Tan**	2016	129		13	354	13	3.7			
**Tejada-Artigas**	2001	130	Cohort	12	103	11	10.7			
**Thompson**	2008	131		6.2	.	.	.	4270	74	1.7
**Timsit**	1996	132	Cohort	19	387	18	4.7			
**Timsit_Fungibact**	2019	133	Cohort	24	213	13	6.1			
**Torres**	1990	134	Cohort	4	322	2	0.6			
**Trouillet**	1998	135	Cohort	20	498	52	10.4			
**Urli**	2002	136	Cohort	21	178	40	22.5	178	12	6.7
**Valles**	2007	137	Cohort	22	60	9	15.0			
**Vanhems**	2011	138	Cohort	6	3387	137	4.0			
**Verhamme**	2007	139	Cohort	8	4000	56	1.4			
**Violan**	1998	140	Cohort	16	314	26	8.3			
**Warren**	2001	141		4.1	3163		.	920	3	0.33
**Woske**	2001	142	Cohort	19	103	29	28.2			
**Xie**	2011	143		7	4155	92	2.2			
**Zahar**	2009	144	Cohort	10	1233	51	4.1			
**Apisarnthanarak**	2007	145		8	952	2	0.2			
**Apisarnthanarak**	2007	145		8	482	2	0.4			
**Apisarnthanarak**	2007	145		8	470	7	1.5			

Table S1 footnotes

Table S2: Studies of non-decontamination-based methods of VAP prevention [a]

Author	Year	Ref	Notes	LOS	Patients (n)	V- S aureus (n)	V- S aureus %	Patients (n)	B- S aureus (n)	B- S aureus %
**Francois_Saatellite**	2021	146	control	21	100	26	26.0	100	7	7.0
**Francois_Saatellite**	2021	146	suvratoxumab	19	196	43	21.9	196	10	5.1
**Acosta-escribano**	2010	147	Gastric feeding	18	54	4	7.4			
**Acosta-escribano**	2010	147	SB feeding	16	50	8	16.0			
**Bonten**	1995	148	Suc	17	67	4	6.0			
**Bonten**	1995	148	Atc	19	74	7	9.5			
**Cook**	1998	149	H2RA	13	596	44	7.4			
**Cook**	1998	149	Suc	14	604	36	6.0			
**Driks**	1987	150	antacid	14	69	4	5.8			
**Driks**	1987	150	sucrulfate	11	61	0	0.0			
**Forestier**	2008	151	uc	13	106	11	10.4			
**Forestier**	2008	151	pbtic	13	102	12	11.8			
**Giamarellos-Bourboulis**	2009	152	probiotic	15				36	1	2.8
**Giamarellos-Bourboulis**	2009	152	no probiotic	15				36	0	0.0
**Heyland**	1999	153	C	12	46	0	0.0			
**Heyland**	1999	153	acid feed	13	49	1	2.0			
**Knight**	2009	154	no probiotic	7	129	1	0.8			
**Knight**	2009	154	probiotic	6	130	0	0.0			
**Kostadima**	2005	155	CC	30	21	3	14.3			
**Kostadima**	2005	155	PEG	30	20					
**Laggner**	1989	156	H2RA	10	16	0	0.0	16	0	0.0
**Laggner**	1989	156	Suc	12	16	0	0.0	16	1	6.3
**Martin**	1993	157	C	10	66	1	1.5			
**Martin**	1993	157	H2RA	10	65	0	0.0			
**Miano**	2009	158	ppi	4	377	3	0.8			
**Miano**	2009	158	ranitidine	4	457	0	0.0			
**Montecalvo**	1992	159	Gastric feeding	10				19	1	5.3
**Montecalvo**	1992	159	SB feeding	10				19	2	10.5
**Morrow**	2010	160	no probiotic	15	73	14	19.2			
**Morrow**	2010	160	probiotic	15	73	8	11.0			
**Pickworth**	1993	161	H2RA	7	44	1	2.3			
**Pickworth**	1993	161	Suc	7	39	0	0			

Table S2 (continued): Studies of non-decontamination-based methods of VAP prevention [a]

Author	Year	Ref	Notes	LOS	Patients (n)	V- S aureus (n)	V- S aureus %	Patients (n)	B- S aureus (n)	B- S aureus %
**Prod'hom_A**	1994	162	No ranitidine	6	81	5	6.2			
**Prod'hom_S**	1994	162	No ranitidine	5	83	2	2.4			
**Prod'hom_R**	1994	162	No sucralfate	5	80	4	5.0			
**Reigneir**	2013	163	no RGV	10	222	17	7.7			
**Reigneir**	2013	163	RGV	10	227	10	4.4			
**Ryan_C**	1993	164	H2RA	5	56	1	1.8			
**Ryan_S**	1993	164	Suc	6	58	2	3.4			
**Zeng**	2016	165	no probiotic	22	117	16	13.7			
**Zeng**	2016	165	probiotic	18	118	12	10.2			
**Damas**	2022	166		11	155	3	1.9			
**Damas**	2022	166	alloy ETT	11	168	1	0.6			
**Dat**	2022	167		19	301	6	2.0			
**Dat**	2022	167	cuff	20	296	7	2.4			
**Djedaini**	1995	168	HME 24	10	61	0	0.0			
**Djedaini**	1995	168	HME48	9	68	2	2.9			
**Drakulovic**	1999	169	supine	10	47	4	8.5			
**Drakulovic**	1999	169	semirecumb	9	39	0	0.0			
**Dreyfuss**	1991	170	cc48	10	35	2	5.7			
**Dreyfuss**	1991	170	cc0	13	28	1	3.6			
**Dreyfuss**	1995	171	HH	10	70	2	2.9			
**Dreyfuss**	1995	171	HME	13	61	0	0.0			
**Holzapfel**	1999	172	no sinus CT	15	200	21	10.5	200	2	1.0
**Holzapfel**	1999	172	sinus CT	17	199	7	3.5	199	2	1.0
**Kirton**	1997	173	HH	17	140	6	4.3			
**Kirton**	1997	173	HME	20	140	6	4.3			
**Kollef '97_post**	1997	174	Cohort	4	327	4	1.2	327	1	0.3
**Kollef '97_pre**	1997	174	Cohort	4	353	5	1.4	353	5	1.4
**Kollef'08**	2008	175	CCAS	4	743	16	2.2			
**Kollef'08**	2008	175	silver	4	766	9	1.2			
**Lacherade**	2005	176	HH	25	184	16	8.7			
**Lacherade**	2005	176	HME	21	185	18	9.7			
**Lacherade**	2010	177	no SSD	11	164	8	4.9			
**Lacherade**	2010	177	SSD	11	169	2	1.2			
**Laueny**	2014	178	control	17	91	6	6.6			
**Laueny**	2014	178	fever control	11	98	17	17.3			

Table S2(continued): Studies of non-decontamination-based methods of VAP prevention [a]

Author	Year	Ref	Notes	LOS	Patients (n)	V- S aureus (n)	V- S aureus %	Patients (n)	B- S aureus (n)	B- S aureus %
**Lorente**	2003	179	WO filter	18	116	8	6.9			
**Lorente**	2003	179	W filter	16	114	7	6.1			
**Lorente**	2004	180	cc48	16	143	6	4.2			
**Lorente**	2004	180	cc0	20	161	14	8.7			
**Lorente**	2006	181	OTSS	13	233	11	4.7			
**Lorente**	2006	181	CTSS	13	210	10	4.8			
**Lorente**	2005	182	OTSS	10	221	8	3.6			
**Lorente**	2005	182	CTSS	10	236	8	3.4			
**Lorente'06b**	2006	183	HH	21	51	5	9.8			
**Lorente'06b**	2006	183	HME	20	53	2	3.8			
**Lorente'07**	2007	184	no SSD	16	140	8	5.7			
**Lorente'07**	2007	184	SSD	14	140	2	1.4			
**Lorente'14**	2014	185	no CPC	16	150	5	3.3			
**Lorente'14**	2014	185	CPC	15	134	1	0.7			
**Mahmoodpoor**	2017	186		18	138	4	2.9			
**Mahmoodpoor**	2017	186	EVAC	15	138	1	0.7			
**Manzano**	2008	187	CC	12	63	9	14.3			
**Manzano**	2008	187	PEEP	9	64	4	6.3			
**Marjanović _AGATE**	2021	188	SC	14	218	27	12.4			
**Marjanović _AGATE**	2021	188	CTSS	14	216	21	9.7			
**Nseir**	2011	189	tr cuff no	10	61	3	4.9			
**Nseir**	2011	189	tr cuff yes	12	61	1	1.6			
**Pneumatikos**	2006	190	CCP_TAP	16	40	4	10.0			
**Pneumatikos**	2006	190	AS	15	39	2	5.1			
**Rumbak**	2004	191	prolonged ETT	5	60	1	1.7			
**Rumbak**	2004	191	prolonged ETT	16	60	5	8.3			
**Smulders**	2002	192	no SSD	14	75	3	4.0			
**Smulders**	2002	192	SSD	12	75	1	1.3			
**Staudinger**	2010	193	no kinetic bed	14	75	2	2.7			
**Staudinger**	2010	193	kinetic bed	8	75	2	2.7			
**Thomachot**	1998	194	hyg	12	66	7	10.6			
**Thomachot**	1998	194	hyd	12	70	8	11.4			
**Thomachot**	1999	195	HMEF1	11	77	8	10.4			
**Thomachot**	1999	195	HMEF2	12	63	7	11.1			
**Thomachot**	2002	196	HME1	9	84	7	8.3			
**Thomachot**	2002	196	HME7	9	71	5	7.0			

Table S2(continued): Studies of non-decontamination-based methods of VAP prevention [a]

Author	Year	Ref	Notes	LOS	Patients (n)	V- S aureus (n)	V- S aureus %	Patients (n)	B- S aureus (n)	B- S aureus %
**Valencia**	2007	197	CC	13	69	2	2.9			
**Valencia**	2007	197	cuff presure	13	73	2	2.7			
**Walaszek**	2017	198	no SSD	5	804	4	0.5			
**Walaszek**	2017	198	SSD	5	1003	2	0.2			

Table S2 footnotes

a. Study interventions; histamine 2 receptor antagonist; proton pump inhibitor; feeding by the gastric route; feeding by the small bowel route; open tracheal suction system; closed tracheal suction system; Heated humidifier; heat and moisture exchanger changed every 24 hours; heat and moisture exchanger changed every 48 hours; subglottic secretion drainage; no circuit changes; circuit changes at 48 hours.

Initial data sources

Alkhawaja S, Martin C, Butler RJ, Gwadry-Sridhar F. Post-pyloric versus gastric tube feeding for preventing pneumonia and improving nutritional outcomes in critically ill adults. Cochrane Database of Systematic Reviews. 2015(8).
Bo L, Li J, Tao T, Bai Y, Ye X, Hotchkiss RS, Kollef MH, Crooks NH, Deng X. Probiotics for preventing ventilator-associated pneumonia. Cochrane Database of Systematic Reviews 2014, Issue 10. Art. No.: CD009066.
Gillies D, Todd DA, Foster JP, Batuwitage BT. Heat and moisture exchangers versus heated humidifiers for mechanically ventilated adults and children. Cochrane Database of Systematic Reviews 2017, Issue 9. Art. No.: CD004711.
Solà I, Benito S. Closed tracheal suction systems versus open tracheal suction systems for mechanically ventilated adult patients. Cochrane Database of Systematic Reviews 2007, Issue 4. Art. No.: CD004581.
Tokmaji G, Vermeulen H, Müller MCA, Kwakman PHS, Schultz MJ, Zaat SAJ. Silver-coated endotracheal tubes for prevention of ventilator-associated pneumonia in critically ill patients. Cochrane Database of Systematic Reviews 2015, Issue 8. Art. No.: CD009201
Toews_I, George_AT, Peter_JV, Kirubakaran_R, Fontes_LES, Ezekiel_JPB, Meerpohl_JJ. Interventions for preventing upper gastrointestinal bleeding in people admitted to intensive care units. *Cochrane Database of Systematic Reviews* 2018, Issue 6. Art. No.: CD008687.
Wang L, Li X, Yang Z, Tang X, Yuan Q, Deng L, Sun X. Semi-recumbent position versus supine position for the prevention of ventilator-associated pneumonia in adults requiring mechanical ventilation. Cochrane Database of Systematic Reviews 2016, Issue 1. Art. No.: CD009946.

Table S3: Studies of topical antiseptic based methods of VAP prevention [a]

Author	Year	Ref	Notes	LOS	Patients (n)	V- S aureus (n)	V- S aureus %	Patients (n)	B- S aureus (n)	B- S aureus %
**Bleasdale**	2007	199	UC	5	445			445	1	0.2
**Bleasdale**	2007	199	CHLX_BW	6	391			391	0	0.0
**Cabov**	2010	200	plac	6	30	2	6.7	30	2	6.7
**Cabov**	2010	200	CHLX	6	30	0	0.0	30	0	0.0
**Caruso**	2009	201	nil	17	132	5	3.8			
**Caruso**	2009	201	saline	17	130	1	0.8			
**Climo_CRT**	2013	202	NCC	6				1398	8	0.6
**Climo_CRT**	2013	202	CHLX_BW	6				1410	9	0.6
**Fourrier'00**	2000	203	UC	24	30	3	10.0	30	1	3.3
**Fourrier'00**	2000	203	CHLX	18	30	0	0.0	30	0	0.0
**Fourrier'05**	2005	204	UC	13	114	2	1.8	114	0	0.0
**Fourrier'05**	2005	204	CHLX	14	114	1	0.9	114	1	0.9

Table S3(continued): Studies of topical antiseptic based methods of VAP prevention [a]

Author	Year	Ref	Notes	LOS	Patients (n)	V- S aureus (n)	V- S aureus %	Patients (n)	B- S aureus (n)	B- S aureus %
**Gacouin**	2009	205	CHLX	11	361	21	5.8			
**Genuit**	2001	206	CHLX	14	56	7	12.5			
**Huang_1SC**	2013	207	screen	4				23480	128	0.5
**Huang_1pre**	2013	207	Cohort	4				15816	77	0.5
**Huang_2pre**	2013	207	NCC	4				15218	70	0.5
**Huang_3pre**	2013	207	NCC	4				17356	80	0.5
**Huang_2TD**	2013	207	CHLX_BW	4				24752	106	0.4
**Huang_3UD**	2013	207	CHLX_BW	4				26024	92	0.4
**Huang**	2023	281	Chlx	3				233661	816	0.35
**Huang**	2023	281	Mupirocin	3				214684	764	0.36
**Huang**	2023	281	Chlx	3				185022	950	0.51
**Huang**	2023	281	Chlx	3				168301	815	0.48
**Koeman**	2006	208	UC	13	130	5	3.8			
**Koeman-Ch**	2006	208	CHLX	14	127	2	1.6			
**KoemanChC**	2006	208	TAP	13	128	5	3.9			
**Kollef'06**	2006	209	Plac	14	347	25	7.2			
**Kollef'06**	2006	209	Iseganan	14	362	17	4.7			
**Lorente'12**	2012	210	CHLX	13	219	4	1.8			
**Lorente'12**	2012	210	CHLX + TB	12	217	4	1.8			
**Milstone**	2013	211	NCC	3	1326			2525	4	0.16
**Milstone**	2013	211	CHLX_BW	3	667			2422	3	0.12
**Mori H**	2006	212	NCC	7	414	5	1.2			
**Mori**	2006	212	PVI	8	1248	7	0.6			
**Morris**	2011	213	NCC	6	1460	75	5.1			
**Morris**	2011	213	CHLX	6	501	7	1.4			
**Noto_pre**	2015	214	NCC	4	4852			4852	15	0.3
**Noto_post**	2015	214	CHLX_BW	4	4488			4488	16	0.4
**Paling**	2017	215	CHLX_BW	5	2935	42	1.4			
**Pobo**	2009	216	TB	14	147	9	6.1			
**Roquilly_all**	2011	217	PVI	22	149	32	21.5			
**Sebastian**	2012	218	UC	8	45	0	0.0			
**Sebastian**	2012	218	CHLX	8	41	1	2.4			
**Segers**	2006	219	UC	1	469	6	1.3			
**Segers**	2006	219	CHLX	1	485	1	0.2			
**Seguin – CC**	2006	220	CCAS	19	31	7	22.6			
**Seguin – SC**	2006	220	Plac	14	31	7	22.6			
**Seguin-PVI**	2006	220	PVI	15	36	3	8.3			
**Seguin**	2014	221	UC	16	72	11	15.3			
**Seguin-PVI**	2014	221	PVI	15	78	14	17.9			

Table S3 footnotes

a. Study interventions; chlorhexidine alone; chlorhexidene with toothbrushing; chlorhexidene with mupirocin; saline installation; povidone-iodeine.

Initial data sources

Hua F, Xie H, Worthington HV, Furness S, Zhang Q, Li C. Oral hygiene care for critically ill patients to prevent ventilator-associated pneumonia. Cochrane Database of Systematic Reviews 2016, Issue 10. Art. No.: CD008367.

Zhao T, Wu X, Zhang Q, Li C, Worthington HV, Hua F. Oral hygiene care for critically ill patients to prevent ventilator-associated pneumonia. Cochrane Database of Systematic Reviews 2020, Issue 12. Art. No.: CD008367.

Table S4: Studies of antibiotic based methods of VAP prevention [a]

Author	Year	Ref	Notes	LOS	Patients (n)	V- S aureus (n)	V- S aureus %	Patients (n)	B- S aureus (n)	B- S aureus %
**Beovic**	2003	222	NCC	11	97			97	2	2.1
**Beovic**	2003	222	Cip_Cot	12	81			81	0	0.0
**Bergmans CC**	2001	223	plac	15	78	6	7.7			
**Bergmans NC**	2001	223	NCC	12	61	5	8.2			
**Bergmans**	2001	223	PGV	13	87	3	3.4			
**Biagioni**	2023	224	NCC	10	262	24	9.2			
**Biagioni**	2023	224	PTA	10	86	7	8.1			
**Bonten NC**	1994	225	NCC	16	54	2	3.7			
**Bonten**	1994	225	PTA	13	22	0	0			
**Bos**	2017	226	TAP	7	807	12	1.5			
**Camus '14 NC**	2014	227	NCC	4	925	17	1.8	925	3	0.3
**Camus '14**	2014	227	PTA	4	1022	0	0.0	1022	2	0.2
**David**	2007	228	PTA-Ctx	10	786	9	1.1	786	4	0.5
**De La Court**	2021	229	PTA-Ctx	11				722	7	1.0
**De La Court**	2021	229	PTA-Ctx	11				1236	12	1.0
**de Smet NC**	2009	230	NCC	9				1990	22	1.1
**de Smet NC SDD**	2009	230	PTA-Ctx	9				2045	9	0.4
**de Smet NC SOD**	2009	230	PTA	9				1904	9	0.5
**Garbino'02**	2002	231	PNeV	9	204	8	3.9	204	11	5.4
**Hartenauer NC**	1990	232	Ctx	14	101	8	7.9	101	4	4.0
**Hartenauer**	1990	232	PTA-Ctx	13	99	3	3.0	99	3	3.0
**Hjortrup**	1997	233	CefTNy	14	150	14	9.3	150	2	1.3
**Landelle**	2018	234	NCC	9	291	10	3.4			
**Landelle**	2018	234	PTNy	9	356	4	1.1			
**Landelle**	2018	234	PTNy	8	413	9	2.2			
**Leone**	2002	235	PTA-Cef	12	324	23	7.1			
**Massart_ECMO**	2023	236	NCC	22	172	3	1.7	172	1	0.6
**Massart_ECMO**	2023	236	PGA	24	69	2	2.9	69	0	0.0
**Nardi '90**	1990	237	NCC	13	50	3	6.0			
**Nardi '90**	1990	237	PTA	12	47	3	6.4			
**Nardi PTAM**	2001	238	PTAM	11	119	1	0.8	119	2	1.7
**Nardi PTA**	2001	238	PTA	12	104	9	8.7	104	2	1.9
**Ong SDD**	2015	239	PTA-Ctx	10				3080	10	0.3
**Oostdijk SDD**	2014	240	PTA-Ctx	6				5483	13	0.2
**Oostdijk SOD**	2014	240	PTA	6				5508	32	0.6
**Paling**	2017	214	PTA-Ctx	6	3801	41	1.1			

Table S4(continued): Studies of antibiotic based methods of VAP prevention [a]

Author	Year	Ref	Notes	LOS	Patients (n)	V- S aureus (n)	V- S aureus %	Patients (n)	B- S aureus (n)	B- S aureus %
**Rouby E**	1994	241	E-NCC	18	251	14	5.6			
**Rouby**	1994	241	P	12	347	21	6.1			
**Silvestri'99**	1999	242	PTA-Ctx	9	117	2	1.7	117	8	6.8
**Silvestri'02**	2002	243	TAP		130	21	16.2			
**Silvestri'04**	2004	244	TAP	16	42	8	19.0			
**Silvestri'04 V**	2004	244	TAP	13	42	6	14.3			
**Steffen**	1994	245	PTNy-Ctx	14	127			127	1	0.8
**Stoutenbeek**	1987	246	NCC	14	59	18	30.5	59	7	11.9
**Stoutenbeek ED**	1987	246	PTA	18	42	9	21.4	42	3	7.1
**Stoutenbeek SDD**	1987	246	PTA-Ctx	11	63	3	4.8	63	0	0.0
**SuDDICU**	2022	247	NCC	11				3191	46	1.4
**SuDDICU**	2022	247	PTNy-Ctx	10				2791	27	1.0
**Veelo**	2008	248	PTA-Ctx	17	231	8	3.5			
**Winter CC**	1992	249	CC	8	92	1	1.1			
**Winter NC**	1992	249	NCC	7	84	4	4.8			
**Winter**	1992	249	PTA-Cz	6	91	0	0.0			
**Wittekamp**	2018	250	NCC	10				2251	13	0.6
**Wittekamp PTNy**	2018	250	PTNy	10				2224	12	0.5
**Wittekamp PTNy**	2018	250	PTNy	11				2082	17	0.8
**Wittekamp Chlx**	2018	250	CHLX	10				2108	25	1.2
**Abele-Horn**	1997	251		22	30	5	16.7			
**Abele-Horn**	1997	251	PTA-Ctx	18	58	9	15.5			
**Aerdts**	1991	252		28	39	4	10.3			
**Aerdts**	1991	252	PNoA-Ctx	23	17	0	0.0			
**Blair**	1991	253		8	130	7	5.4			
**Blair**	1991	253	PTA-Ctx	8	126	1	0.8			
**Camus**	2005	254	plac	13	126	8	6.3	126	1	0.8
**Camus PT&MCh**	2005	254	PT_MCh	11	129	1	0.8	129	2	1.6
**Camus '05 PT**	2005	254	PT	12	130	6	4.6	130	3	2.3
**Camus '05 MCh**	2005	254	CHLX	10	130	3	2.3	130	1	0.8
**Cerra**	1992	255	plac	26	21	8	38.1			
**Cerra**	1992	255	NoNy	18	25	5	20.0			
**de la Cal**	2005	256	plac	30	54	16	29.6	54	6	11.1
**de la Cal**	2005	256	PTA-Ctx	30	53	12	22.6	53	14	26.4
**de_Latorre**	1995	257	TAP	15	80	3	3.8			
**Ferrer**	1994	258	Ctx	14	41	2	4.9	41	2	4.9
**Ferrer**	1994	258	PTA-Ctx	15	39	3	7.7	39	0	0.0

Table S4(continued): Studies of antibiotic based methods of VAP prevention [a]

Author	Year	Ref	Notes	LOS	Patients (n)	V- S aureus (n)	V- S aureus %	Patients (n)	B- S aureus (n)	B- S aureus %
**Gastinne**	1992	259	plac	19	225	6	2.7			
**Gastinne**	1992	259	PTA	18	220	15	6.8			
**Gaussorgues**	1991	260		17	59			59	2	3.4
**Gaussorgues**	1991	260	PGA	16	59			59	1	1.7
**Georges**	1994	261		16	33	1	3.0			
**Georges**	1994	261	PNeA	16	31	0	0.0			
**Hammond**	1994	262	Ctx	14	33	3	9.1			
**Hammond**	1994	262	PTA-Ctx	16	39	4	10.3			
**Jacobs**	1992	263		10	43	0	0.0	43	0	0.0
**Jacobs**	1992	263	PTA-Ctx	9	36	0	0.0	36	0	0.0
**Karvouniaris**	2015	264	plac	13	84	4	4.8			
**Karvouniaris**	2015	264	P	16	84	5	6.0			
**Korinek**	1993	265	plac	27	60	16	26.7			
**Korinek**	1993	265	PTA-V	25	63	9	14.3			
**Laggner**	1994	266	PPAP	30	34	0	0.0	34	0	0.0
**Laggner**	1994	266	GA	25	33	0	0.0	33	1	3.0
**Langlois-Karaga**	1995	267	plac	11	50	15	30.0			
**Langlois-Karaga**	1995	267	PGA	11	47	5	10.6			
**Palomar Ctx**	1997	268	Ctx	8	46	3	6.5			
**Palomar**	1997	268		6	42	8	19.0			
**Palomar**	1997	268	PTA-Ctx	11	41	5	12.2			
**Quinio**	1996	269	plac	16	72	16	22.2	72	4	5.6
**Quinio**	1996	269	PGA	16	76	9	11.8	76	8	10.5
**Reizine**	2019	270	PVI	14	149	54	36.2			
**Reizine**	2019	270	PVI-PAP	13	146	36	24.7			
**Rimola**	1985	271		10	72			72	5	6.9
**Rimola**	1985	271	PGVNy	11	68			68	2	2.9
**Rocha**	1992	272	plac	18	54	15	27.8	54	3	5.6
**Rocha**	1992	272	PTA-Ctx	19	47	5	10.6	47	2	4.3
**Rodríguez-Roldán**	1990	273	plac	12	15	1	6.7			
**Rodríguez-Roldán**	1990	273	PTNeA	10	13	0	0.0			
**Rolando_i '93**	1993	274	Cfu	8	21	0	0.0	21	0	0.0
**Rolando '93**	1993	274	PTAMCfu	8	28	2	7.1	28	0	0.0
**Sanchez-Garcia**	1998	275	plac	20	140	7	5.0			
**Sanchez-Garcia**	1998	275	PTA-Ctx	17	131	5	3.8			

Table S4(continued): Studies of antibiotic based methods of VAP prevention [a]

Author	Year	Ref	Notes	LOS	Patients (n)	V- S aureus (n)	V- S aureus %	Patients (n)	B- S aureus (n)	B- S aureus %
**Stoutenbeek**	2007	276		12	200	40	20.0	200	5	2.5
**Stoutenbeek**	2007	276	PTA-Ctx	13	201	18	9.0	201	8	4.0
**Ulrich**	1989	277		13	52	5	9.6			
**Ulrich**	1989	277	PNoA_Tr	17	48	2	4.2			
**Unertl**	1987	278		23	20	5	25.0			
**Unertl**	1987	278	PGA	18	19	1	5.3			
**Verwaest**	1997	279		19	185	9	4.9	185	3	1.6
**Verwaest OA**	1997	279	OfA-Of	17	193	6	3.1	193	7	3.6
**Verwaest PTA**	1997	279	PTA-Ctx	22	200	9	4.5	200	10	5.0
**Wiener**	1995	280	plac	11	31	4	12.9			
**Wiener**	1995	280	PGNy	11	30	1	3.3			

Footnotes

a. Treatment abbreviations

- Topical antibiotic components include; topical polymyxin; topical tobramycin; topical amphotericin; topical gentamicin; topical ciprofloxacin, topical vancomycin; topical nystatin; topical neomycin; topical nalidixic acid; topical norfloxacin; topical oflaxcin; nebulized polymyxin; nebulized saline.
- Parenteral antibiotic components include; Amoycillin-clavulinate; systemic ampicillin; systemic Cefuroxime; systemic cefotaxime; systemic ciprofloxacin; systemic vancomycin; systemic ciprofloxacin; systemic trimethoprim; systemic oflaxcin; systemic ceftazidime.

Initial data sources

Liberati A, D'Amico R, Pifferi S, Torri V, Brazzi L, Parmelli E. Antibiotic prophylaxis to reduce respiratory tract infections and mortality in adults receiving intensive care. Cochrane Database of Systematic Reviews 2009, Issue 4. Art. No.: CD000022.

Minozzi S, Pieri S, Brazzi L, Pecoraro V, Montrucchio G, D'Amico R. Topical antibiotic prophylaxis to reduce respiratory tract infections and mortality in adults receiving mechanical ventilation. Cochrane Database of Systematic Reviews 2021, Issue 1. Art. No.: CD000022.

Table S5. Meta-regression models of prevention effect size versus LOS [a]

	S aureus VAP			S aureus bacteremia		
	coefficient	95% CI;	n	coefficient	95% CI;	n
Non-decontamination						
• Intercept	-0.41	-0.69 to -0.12	51	-0.95	-2.23 to +0.32	5
• Slope	+0.22	-0.16 to +0.6		+0.78	-0.72 to +2.28	
Anti-septic						
• Intercept	-1.18	-1.66 to -0.69	13	+0.26	-0.11 to +0.63	10
• Slope	+0.9	+0.07 to +1.72		+0.72	+0.04 to +1.4	
Antibiotic						
• Intercept	-1.02	-1.56 to -0.48	41	-0.8	-1.2 to -0.38	25
• Slope	+0.57	-0.09 to +1.22		+1.26	+0.59 to +1.93	

Footnotes to table S5

a. Note, several studies had more than one control and or intervention group and hence contribute more than once to the study count.
b. Non-decontamination studies see Figure S4 (S aureus VAP prevention) & S5 (S aureus BSI prevention)
c. Anti-septic studies; see Figure S6 (S aureus VAP prevention) & S7 (S aureus BSI prevention)
d. Antibiotic studies; see Figure S8 (S aureus VAP prevention) & S9 (S aureus BSI prevention)

Table S6. Meta-regression models of *S aureus* infection incidence versus LOS [a]

	Intercept (at day 7)		Slope	
	coefficient	95% CI;	coefficient	95% CI;
S aureus VAP				
Observational (benchmark)	-3.47 [b]	-3.6 to -3.29	+0.9 [b]	+0.6 to +1.2
Non concurrent control	0.24	-0.35 to +0.83	0.21	-0.83 to + 1.24
Non-decontamination Control	0.08	-0.35 to +0.5	0.12	-0.49 to + 0.74
Non-decontamination Intervention	-0.26	-0.71 to +0.2	0.26	-0.41 to +0.95
Anti-septic Control	-0.05	-0.92 to +0.82	0.62	-0.52 to +1.76
Anti-septic Intervention	-0.82	-1.5 to -0.17	1.02	+0.01 to +2.05
Antibiotic Control	0.83	+0.1 to +1.56	-0.26 [b]	-1.1 to +0.61
Antibiotic Intervention	-0.6	-1.1 to -0.09	0.88 [b]	+0.17 to +1.6
S aureus BSI				
Observational (benchmark)	-4.48 [c]	-4.74 to -4.2	+1.01 [c]	+0.54 to +1.47
Non concurrent control	-0.41	-0.98 to +0.17	0.79	-0.28 to + 1.86
Non-decontamination Control	0.62	-0.27 to +1.51	-0.89	-2.07 to +0.3
Non-decontamination Intervention	-0.06	-1.36 to + 1.25	-0.16	-1.8 to +1.48
Anti-septic Control	-0.03	-0.89 to +0.82	0.24	-1.18 to +1.61
Anti-septic Intervention	-0.53	-1.19 to +0.12	0.33	-0.97 to +1.62
Antibiotic Control	0.4	-0.9 to +1.71	-0.25 [c]	-1.75 to +1.3
Antibiotic Intervention	-0.61	-1.13 to -0.08	1.27 [c]	+0.44 to +2.09

Footnotes to table S6

a. Interpretation, the observational group is the benchmark group, the coefficients for the other groups represent the increment additional to the benchmark group.

b. Contrast between slope coefficients for *S aureus* VAP incidence slopes versus lnLOS for control versus intervention groups was -1.14 (-2.2 to -0.1; p = 0.032). Other contrast for *S aureus* VAP incidence slopes for control versus intervention groups were non-significant.

c. Contrast between slope coefficients for *S aureus* BSI incidence slopes versus lnLOS for control versus intervention groups was -1.51 (-3.1 to +0.06; p = 0.059). Other contrast for *S aureus* BSI incidence slopes for control versus intervention groups were non-significant.

References

S1. A'Court CH, Garrard CS, Crook D, Bowler I, Conlon C, Peto T, Anderson E: Microbiological lung surveillance in mechanically ventilated patients, using non-directed bronchial lavage and quantitative culture. *Q J Med.* 1993;86:635-48.

S2. Alvarez-Lerma F, ICU-acquired Pneumonia Study Group. Modification of empiric antibiotic treatment in patients with pneumonia acquired in the intensive care unit. *Intens Care Med.* 1996;22(5):387-94.

S3. Apostolopoulou E, Bakakos P, Katostaras T, Gregorakos L: Incidence and risk factors for ventilator-associated pneumonia in 4 multidisciplinary intensive care units in Athens, Greece. *Respir Care*. 2003;48: 681-688.

S4. Arroliga AC, Pollard CL, Wilde CD, Pellizzari SJ, Chebbo A, Song J, Ordner J, et al Reduction in the incidence of ventilator-associated pneumonia: a multidisciplinary approach. *Respiratory care*. 2012;57(5):688-96.

S5. Arumugam SK, Mudali I, Strandvik G, El-Menyar A, Al-Hassani A, Al-Thani H. Risk factors for ventilator-associated pneumonia in trauma patients: A descriptive analysis. *World J Emerg Med*. 2018;9(3):203.

S6. Baldesi O, Bailly S, Ruckly S, Lepape A, L'Heriteau F, et al. ICU-acquired candidaemia in France: epidemiology and temporal trends, 2004–2013–a study from the REA-RAISIN network. *J Infect.* 2017;75(1):59-67.

S7. Bekaert M, Timsit JF, Vansteelandt S, Depuydt P, Vésin A, Garrouste-Orgeas M, Decruyenaere J, Clec'h C, Azoulay E, Benoit D. Attributable mortality of ventilator-associated pneumonia: a reappraisal using causal analysis. *Am J Respir Crit Care Med*. 2011;184(10):1133-9.

S8. Bercault N, Boulain T: Mortality rate attributable to ventilator-associated nosocomial pneumonia in an adult intensive care unit: a prospective case-control study. *Crit Care Med*. 2001;29:2303-2309

S9. Blot S, Koulenti D, Dimopoulos G, Martin C, Komnos A, Krueger WA, Spina G, Armaganidis A, Rello J. Prevalence, risk factors, and mortality for ventilator-associated pneumonia in middle-aged, old, and very old critically ill patients. *Crit Care Med*. 2014;42(3):601-9.

S10. Bochicchio GV, Joshi M, Bochicchio K, Tracy K, Scalea TM: A time-dependent analysis of intensive care unit pneumonia in trauma patients. *J Trauma*. 2004;56:296-301.

S11. Bonten MJ, Gaillard CA, van Tiel FH, Smeets HG, van der Geest S, Stobberingh EE: The stomach is not a source for colonization of the upper respiratory tract and pneumonia in ICU patients. *Chest*. 1994;105(3):878-84.

S12. Boots RJ, George N, Faoagali JL, Druery J, Dean K, Heller RF. Double-heater-wire circuits and heat-and-moisture exchangers and the risk of ventilator-associated pneumonia. *Crit Care Med*. 2006 ;34(3):687-93.

S13. Boots RJ, Phillips GE, George N, Faoagali JL: Surveillance culture utility and safety using low-volume blind bronchoalveolar lavage in the diagnosis of ventilator-associated pneumonia. *Respirology*. 2008;13:87-96

S14. Bornstain C, Azoulay E, De Lassence A, Cohen Y, Costa MA, Mourvillier B, Descorps-Declere A, Garrouste-Orgeas M, Thuong M, Schlemmer B, Timsit JF: Sedation, sucralfate, and antibiotic use are potential means for protection against early-onset ventilator-associated pneumonia. *Clin Infect Dis*. 2004;38(10):1401-8.

S15. Braun SR, Levin AB, Clark KL. Role of corticosteroids in the development of pneumonia in mechanically ventilated head-trauma victims. *Crit Care Med* 1986;14:198-201

S16. Bregeon F, Papazian L, Visconti A, Gregoire R, Thirion X, Gouin F: Relationship of microbiologic diagnostic criteria to morbidity and mortality in patients with ventilator-associated pneumonia. *JAMA*. 1997;277: 655-662

S17. Cade JF, McOwat E, Siganporia R, Keighley C, Presneill J, Sinickas V: Uncertain relevance of gastric colonization in the seriously ill. *Intensive Care Med*. 1992;18:210-217

S18. Cavalcanti M, Ferrer M, Ferrer R, Morforte R, Garnacho A, Torres A: Risk and prognostic factors of ventilator-associated pneumonia in trauma patients. *Crit Care Med*. 2006;34:1067-1072

S19. Cendrero JA, Solé-Violán J, Benitez AB, Catalán JN, Fernández JA, Santana PS, de Castro FR: Role of different routes of tracheal colonization in the development of pneumonia in patients receiving mechanical ventilation. *Chest*. 1999;116:462-470

S20. Chaari A, El Habib M, Ghdhoun H, Algia NB, Chtara K, Hamida CB, Chelly H, Bahloul M, Bouaziz M. Does low-dose hydrocortisone therapy prevent ventilator-associated pneumonia in trauma patients? *Am J Therap*. 2015;22(1):22-8.

S21. Chastre J, Trouillet JL, Vuagnat A, Joly-Guillou ML, Clavier H, Dombret MC, Gibert C: Nosocomial pneumonia in patients with acute respiratory distress syndrome. *Am J Respir Crit Care Med*. 1998;157:1165-1172

S22. Chevret S, Hemmer M, Carlet J: Incidence and risk factors of pneumonia acquired in intensive care units. Results from a multicenter prospective study on 996 patients. European Cooperative Group on Nosocomial Pneumonia. *Intensive Care Med*. 1993;19:256-264

S23. Combes P, Fauvage B, Oleyer C. Nosocomial pneumonia in mechanically ventilated patients, a prospective randomised evaluation of the Stericath closed suctioning system. *Intensive Care Med* 2000;26:878-82.

S24. Cook A, Norwood S, Berne J: Ventilator-associated pneumonia is more common and of less consequence in trauma patients compared with other critically ill patients. *J Trauma Acute Care Surg*. 2010;69(5):1083-91.

S25. Craven DE, Kunches LM, Lichtenberg DA, Kollisch NR, Barry MA, Heeren TC, McCabe WR: Nosocomial infection and fatality in medical and surgical intensive care unit patients. *Arch Intern Med.* 1988;148:1161-1168

S26. Daschner F, Kappstein I, Schuster F, Scholz R, Bauer E, Jooβens D, Just H: Influence of disposable ('Conchapak') and reusable humidifying systems on the incidence of ventilation pneumonia. J Hosp Infect. 1988;11:161-168

S27. Delclaux C, Roupie E, Blot F, Brochard L, Lemaire F, Brun-Buisson C. Lower respiratory tract colonization and infection during severe acute respiratory distress syndrome: incidence and diagnosis. *Am J Respir Crit Care Med*. 1997;156(4):1092-8.

S28. Delle Rose D, Pezzotti P, Fortunato E, Sordillo P, Gini S, Boros S, Meledandri M, Gallo MT, et al. Clinical predictors and microbiology of ventilator-associated pneumonia in the intensive care unit: a retrospective analysis in six Italian hospitals. *Eur J Clin Microbiol & Infect Dis*. 2016;35:1531-9.

S29. Edgeworth JD, Treacher DF, Eykyn SJ. A 25-year study of nosocomial bacteremia in an adult intensive care unit. *Crit Care Med*. 1999 ;27(8):1421-8.

S30. El-Masri MM, Hammad TA, McLeskey SW, Joshi M, et al Predictors of nosocomial bloodstream infections among critically ill adult trauma patients. *Infect Cont & Hosp Epidemiol.* 2004;25(8):656-63.

S31. Ensminger SA, Wright RS, Baddour LM, Afess B: Suspected ventilator-associated pneumonia in cardiac patients admitted to the coronary care unit. *Mayo Clin Proc*. 2006;81:32–35

S32. Ertugrul BM, Yildirim A, Ay P, Oncu S, Cagatay A, Cakar N, Ertekin C, Ozsut H, Eraksoy H, Calangu S. Ventilator-associated pneumonia in surgical emergency intensive care unit. *Saudi Med J*. 2006;27(1):52-7.

S33. Esnault P, Nguyen C, Bordes J, D'Aranda E, Montcriol A, Contargyris C, Cotte J, et al. Early-onset ventilator-associated pneumonia in patients with severe traumatic brain injury: incidence, risk factors, and consequences in cerebral oxygenation and outcome. *Neurocritical Care*. 2017;27:187-98.

S34. Evans HL, Zonies DH, Warner KJ, Bulger EM, Sharar SR, Maier RV, Cuschieri J. Timing of intubation and ventilator-associated pneumonia following injury. *Arch Surg*. 2010;145(11):1041-6.

S35. Ewig S, Torres A, El-Ebiary M, Fàbregas N, Hernandez C, Gonzalez J, Nicolas JM, Soto L: Bacterial colonization patterns in mechanically ventilated patients with traumatic and medical head injury. Incidence, risk factors, and association with ventilator-associated pneumonia. *Am J Respir Crit Care Med*. 1999;159:188-198

S36. Fabian TC, Boucher BA, Croce MA, Kuhl DA, Janning SW, Coffey BC, Kudsk KA: Pneumonia and stress ulceration in severely injured patients: a prospective evaluation of the effects of stress ulcer prophylaxis. *Arch Surg*. 1993;128(2):185-92.

S37. Fagon JY, Chastre J, Domart Y, Trouillet JL, Pierre J, Darne C, Gibert C: Nosocomial pneumonia in patients receiving continuous mechanical ventilation. Prospective analysis of 52 episodes with use of a protected specimen brush and quantitative culture techniques. *Am Rev Respir Dis* 1989;139:877-884.

S38. García-Garmendia JL, Ortiz-Leyba C, Garnacho-Montero J, Jiménez-Jiménez FJ, et al. Risk factors for *Acinetobacter baumannii* nosocomial bacteremia in critically ill patients: a cohort study. *Clin Infect Dis*. 2001 ;33(7):939-46.

S39. Garrouste-Orgeas M, Chevret S, Arlet G, Marie O, Rouveau M, Popoff N, Schlemmer B: Oropharyngeal or gastric colonization and nosocomial pneumonia in adult intensive care unit patients. A prospective study based on genomic DNA analysis. *Am J Respir Crit Care Med.* 1997;156(5):1647-56.

S40. Garrouste-Orgeas M, Timsit JF, Tafflet M, Misset B, Zahar JR, et al: Excess risk of death from intensive care unit—acquired nosocomial bloodstream infections: a reappraisal. *Clin Infect Dis* 2006, 42:1118-1126.

S41. George DL, Falk PS, Wunderink RG, Leeper Jr KV, Meduri GU, Steere EL, Glen Mayhall C: Epidemiology of ventilator-acquired pneumonia based on protected bronchoscopic sampling. *Am J Respir Crit Care Med*. 1998;158:1839-1847

S42. Georges H, Leroy O, Guery B, Alfandari S, Beaucaire G: Predisposing factors for nosocomial pneumonia in patients receiving mechanical ventilation and requiring tracheotomy. *Chest*. 2000;118:767–774.

S43. Giard M, Lepape A, Allaouchiche B, Guerin C, Lehot JJ, Robert MO, Vanhems P: Early-and late-onset ventilator-associated pneumonia acquired in the intensive care unit: comparison of risk factors. *J Crit Care* 2008, 23:27-33.

S44. Girou E, Schortgen F, Delclaux C, Brun-Buisson C, Blot F, et al. Association of noninvasive ventilation with nosocomial infections and survival in critically ill patients. *JAMA*. 2000;284(18):2361-7.

S45. Gouel-Cheron A, Swihart BJ, Warner S, Mathew L, Strich JR, Mancera A, Follmann D, Kadri SS. Epidemiology of ICU-Onset Bloodstream Infection: Prevalence, Pathogens, and Risk Factors Among 150,948 ICU Patients at 85 US Hospitals. *Crit Care Med*. 2022;50(12):1725-36.

S46. Gruson D, Hilbert G, Vargas F, Valentino R, Bebear C, Allery A, Bebear C, Gbikpi-Benissan GE, Cardinaud JP: Rotation and restricted use of antibiotics in a medical intensive care unit: impact on the incidence of ventilator-associated pneumonia caused by antibiotic-resistant gram-negative bacteria. *Am J Respir Crit Care Med*. 2000, 162(3):837-43.

S47. Gruson D, Hilbert G, Vargas F, Valentino R, Bui N, Pereyre S, Bebear C, Bebear CM, Gbikpi-Benissan G: Strategy of antibiotic rotation: long-term effect on incidence and susceptibilities of Gram-negative bacilli responsible for ventilator-associated pneumonia. *Crit Care Med*. 2003;31:1908-1914.

S48. Guérin C, Girard R, Chemorin C, De Varax R, Fournier G: Facial mask noninvasive mechanical ventilation reduces the incidence of nosocomial pneumonia. *Intens care Med.* 1997;23(10):1024-32.

S49. Guimaraes MM, Rocco JR: Prevalence of ventilator-associated pneumonia in a university hospital and prognosis for the patients affected. *J Bras Pneumol* 2006;32:339–346.

S50. Gursel G, Aydogdu M, Nadir Ozis T, Tasyurek S. Comparison of the value of initial and serial endotracheal aspirate surveillance cultures in predicting the causative pathogen of ventilator-associated pneumonia. *Scandinavian J Infect Dis* 2010;42:341-346

S51. Heyland DK, Cook DJ, Schoenfeld PS, Frietag A, Varon J, Wood G: The effect of acidified enteral feeds on gastric colonization in critically ill patients: results of a multicenter randomized trial. Canadian Critical Care Trials Group. *Crit Care Med*. 1999;27:2399-2406

S52. Holzapfel L, Chevret S, Madinier G, Ohen F, Demingeon G, Coupry A, Chaudet M: Influence of long-term oro- or nasotracheal intubation on nosocomial maxillary sinusitis and pneumonia: results of a prospective, randomized, clinical trial. *Crit Care Med.* 1993;21:1132-1138

S53. Hortal J, Muñoz P, Cuerpo G, Litvan H, Rosseel PM, Bouza E, European Study Group on Nosocomial Infections, European Workgroup of Cardiothoracic Intensivists. Ventilator-associated pneumonia in patients undergoing major heart surgery: an incidence study in Europe. Critical care. 2009;13:1-0.

S54. Hugonnet S, Uçkay I, Pittet D Staffing level: a determinant of late-onset ventilator-associated pneumonia. *Crit Care*. 2007;11(4):R80

S55. Hyllienmark P, Gardlund B, Persson JO, Ekdahl K. Nosocomial pneumonia in the ICU: a prospective cohort study. *Scand J Infect Dis.* 2007;39:676-82.

S56. Ibáñez J, Peñafiel A, Marsé P, Jordá R, Raurich JM, Mata F: Incidence of gastroesophageal reflux and aspiration in mechanically ventilated patients using small-bore nasogastric tubes. *J Parenteral and Enteral Nutrition*. 2000;24(2):103-6.

S57. Ibrahim EH, Ward S, Sherman G, Kollef MH: A comparative analysis of patients with early-onset vs late-onset nosocomial pneumonia in the ICU setting. *Chest*. 2000;117:1434-1442

S58. Ibrahim EH, Sherman G, Ward S, Fraser VJ, Kollef MH. The influence of inadequate antimicrobial treatment of bloodstream infections on patient outcomes in the ICU setting. *Chest*. 2000;118(1):146-55.

S59. Jacobs S, Chang RW, Lee B, Bartlett FW: Continuous enteral feeding: a major cause of pneumonia among ventilated intensive care unit patients. *JPEN J Parenter Enteral Nutr* 1990;14:353-6.

S60. Jaillette E, Nseir S: Relationship between inhaled β2-agonists and ventilator-associated pneumonia: A cohort study. *Crit Care Med*. 2011;39(4):725-30.

S61. Jaimes F, De La Rosa G, Gómez E, Múnera P, Ramírez J, et al. Incidence and risk factors for ventilator-associated pneumonia in a developing country Where is the difference? *Respir Med*. 2007;101:762–767.

S62. Jiménez P, Torres A, Rodríguez-Roisin R, de la Bellacasa JP, Aznar R, Gatell JM, Agustí-Vidal A: Incidence and etiology of pneumonia acquired during mechanical ventilation. *Crit Care Med*. 1989;17:882-5.

S63. Kallel H, Chelly H, Bahloul M, Ksibi H, Dammak H, Chaari A, Hamida CB, Rekik N, Bouaziz M. The effect of ventilator-associated pneumonia on the prognosis of head trauma patients. *J Trauma Acute Care Surg*. 2005;59(3):705-10.

S64. Kallel H, Houcke S, Resiere D, Roy M, Mayence C, Mathien C, Mootien J, Demar M, Hommel D, Djossou F. Epidemiology and Prognosis of Intensive Care Unit–Acquired Bloodstream Infection. *Am J Trop med & Hygiene*. 2020;103(1):508.

S65. Kanafani ZA, Kara L, Hayek S, et al. Ventilator-associated pneumonia at a tertiary-care center in a developing country: incidence, microbiology, and susceptibility patterns of isolated microorganisms. *Infect Control Hosp Epidemiol*. 2003;24:864-9.

S66. Kantorova I, Svoboda P, Scheer P, Doubek J, Rehorkova D, Bosakova H, Ochmann J. Stress ulcer prophylaxis in critically ill patients: a randomized controlled trial. Hepato-gastroenterology. 2004;51(57):757-61.

S67. Karin A, Peršec J, Bakran K, Pražetina M, Šribar A. Etiology, incidence and mortality in patients with ventilator-associated pneumonia in adult general surgery and cardiac surgery intensive care units in University Hospital Dubrava. *Infektološki glasnik*. 2019;39(4):124-8.

S68. Kasuya Y, Hargett JL, Lenhardt R, Heine MF, Doufas AG, Remmel KS, Ramirez JA, Akça O. Ventilator-associated pneumonia in critically ill stroke patients: frequency, risk factors, and outcomes. *J critical care.* 2011;26(3):273-9.

S69. Kirschenbaum L, Azzi E, Sfeir T, et al. Effect of continuous lateral rotational therapy on the prevalence of ventilator-associated pneumonia in patients requiring long-term ventilatory care *Crit Care Med* 2002;30:1983-6.

S70. Ko HK, Yu WK, Lien TC, Wang JH, Slutsky AS, Zhang H, Kou YR. Intensive care unit-acquired bacteremia in mechanically ventilated patients: clinical features and outcomes. *PloS one*. 2013;8(12):e83298.

S71. Kollef MH: Ventilator-associated pneumonia. A multivariate analysis. *JAMA*. 1993;270:1965-70.

S72. Kollef MH, Shapiro SD, Fraser VJ, Silver P, Murphy DM, Trovillion E, Hearns ML, Richards RD, Cracchilo L, Hossin L: Mechanical ventilation with or without 7-day circuit changes. A randomized controlled trial. *Ann Intern Med*.1995;123:168–174

S73. Kollef MH, Silver P, Murphy DM, Trovillion E: The effect of late-onset ventilator-associated pneumonia in determining patient mortality. *Chest*. 1995;108: 1655-62.

S74. Kollef MH, Von Harz B, Prentice D, Shapiro SD, Silver P, John RS, Trovillion E: Patient transport from intensive care increases the risk of developing ventilator-associated pneumonia. *Chest*. 1997;112(3):765-773.

S75. Kollef MH, Prentice D, Shapiro SD, Fraser VJ, Silver P, Trovillion E, Weilitz P, Von Harz B, ST. JOHN RO. Mechanical ventilation with or without daily changes of in-line suction catheters. *Am J Respir Crit Care Med*. 1997;156(2):466-72.

S76. Kollef MH, Chastre J, Fagon JY, François B, Niederman MS, Rello J, Torres A, Vincent JL, Wunderink RG, Go KW, Rehm C. Global prospective epidemiologic and surveillance study of ventilator-associated pneumonia due to Pseudomonas aeruginosa. *Crit Care Med*. 2014;42(10):2178-87.

S77. Kołpa M, Wałaszek M, Gniadek A, Wolak Z, Dobroś W. Incidence, microbiological profile and risk factors of healthcare-associated infections in intensive care units: a 10 year observation in a provincial hospital in Southern Poland. *International J environ res & public health*. 2018;15(1):112.

S78. Koss WG, Khalili TM, Lemus JF, Chelly MM, Margulies DR, Shabot MM: Nosocomial pneumonia is not prevented by protective contact isolation in the surgical intensive care unit. *Am Surg*. 2001;67:1140-4.

S79. Kunac A, Sifri ZC, Mohr AM, Horng H, Lavery RF, Livingston DH: Bacteremia and Ventilator-Associated Pneumonia: A Marker for Contemporaneous Extra-Pulmonic Infection. *Surg Infect.* 2014;15:77-83.

S80. Lambert ML, Suetens C, Savey A, Palomar M, Hiesmayr M, Morales I, Agodi A, Frank U, Mertens K, Schumacher M, Wolkewitz M. Clinical outcomes of health-care-associated infections and antimicrobial resistance in patients admitted to European intensive-care units: a cohort study. *Lancet* Infect Dis. 2011;11(1):30-8.

S81. Laupland KB, Zygun DA, Davies HD, Church DL, Louie TJ, Doig CJ Population-based assessment of intensive care unit-acquired bloodstream infections in adults: incidence, risk factors, and associated mortality rate. *Crit Care Med* 2002;30:2462-2467.

S82. Laupland KB, Kirkpatrick AW, Church DL, Ross T, Gregson DB Intensive-care-unit-acquired bloodstream infections in a regional critically ill population. *J Hosp Infect* 2004;58(2): 137-145.

S83. Leblebicioglu H, Ersoz G, Rosenthal VD, Nevzat-Yalcin A, Akan ÖA, Sirmatel F, Turgut H, Ozdemir D, Alp E, Uzun C, Ulusoy S. Impact of a multidimensional infection control approach on catheter-associated urinary tract infection rates in adult intensive care units in 10 cities of Turkey: International Nosocomial Infection Control Consortium findings (INICC). *Amer J infection control.* 2013;41(10):885-91.

S84. Lepelletier D, Roquilly A, Mahe PJ, Loutrel O, Champin P, Corvec S, Naux E, Pinaud M, Lejus C, Asehnoune K. Retrospective analysis of the risk factors and pathogens associated with early-onset ventilator-associated pneumonia in surgical-ICU head-trauma patients. *J Neurosurg Anesthesiol*. 2010;22(1):32-7.

S85. Lowy FD, Carlisle PS, Adams A, Feiner C. The incidence of nosocomial pneumonia following urgent endotracheal intubation. *Infect Control Hosp Epidemiol*. 1987;8(6):245-8.

S86. Luna CM, Blanzaco D, Niederman MS, et al Resolution of ventilator-associated pneumonia: prospective evaluation of the clinical pulmonary infection score as an early clinical predictor of outcome. *Crit Care Med* 2003;31:676-682

S87. Luyt CE, Guérin V, Combes A, Trouillet JL, Ayed SB, Bernard M, Gibert C, Chastre J: Procalcitonin kinetics as a prognostic marker of ventilator-associated pneumonia. *Am J Respir Crit Care Med*. 2005;171:48-53.

S88. Magnason S, Kristinsson KG, Stefansson T, Erlendsdottir H, Jonsdottir K, Kristjansson M, Gudmundsson S: Risk factors and outcome in ICU-acquired infections. *Acta Anaesthesiologica Scandinavica*. 2008;52:1238-1245

S89. Magret M, Amaya-Villar R, Garnacho J, Lisboa T, Diaz E, DeWaele J, Deja M, Manno E, Rello J, EU-VAP/CAP Study Group: Ventilator-associated pneumonia in trauma patients is associated with lower mortality: results from EU-VAP study. *J Trauma Acute Care Surg*. 2010;69(4):849-854.

S90. Mahul P, Auboyer C, Jospe R, Ros A, Guerin C, el Khouri Z, Galliez M, Dumont A, Gaudin O: Prevention of nosocomial pneumonia in intubated patients respective role of mechanical subglottic secretions drainage and stress ulcer prophylaxis. *Intensive Care Med*. 1992;18:20-25

S91. Makris D, Manoulakas E, Komnos A, Papakrivou E, Tzovaras N, Hovas A, Zintzaras E, Zakynthinos E. Effect of pravastatin on the frequency of ventilator-associated pneumonia and on intensive care unit mortality: open-label, randomized study. *Crit Care Med*. 2011;39(11):2440-6.

S92. Markowicz P, Wolff M, Djedaini K, Cohen Y, Chastre J, Delclaux C: Multicenter prospective study of ventilator-associated pneumonia during acute respiratory distress syndrome. Incidence, prognosis, and risk factors. ARDS Study Group. *Am J Respir Crit Care Med*. 2000;161:1942-8.

S93. Massart N, Wattecamps G, Moriconi M, Fillatre P. Attributable mortality of ICU acquired bloodstream infections: a propensity-score matched analysis. *Eur J Clin Microbiol & Infect Dis.* 2021;40(8):1673-80.

S94. Meduri GU, Reddy RC, Stanley T, El-Zeky F. Pneumonia in acute respiratory distress syndrome: a prospective evaluation of bilateral bronchoscopic sampling. *Am J Respir Crit Care Med*. 1998;158(3):870-5.

S95. Memish ZA, Cunningham G, Oni GA, et al The incidence and risk factors of ventilator-associated pneumonia in a Riyadh hospital. *Infect Control Hosp Epidemiol* 2000;21:271-273

S96. Michel F, Franceschini B, Berger P, Arnal JM, Gainnier M, Sainty JM, Papazian L. Early antibiotic treatment for BAL-confirmed ventilator-associated pneumonia: a role for routine endotracheal aspirate cultures. *Chest*. 2005;127(2):589-97.

S97. Moine P, Timsit JF, De Lassence A, Troché G, Fosse JP, Alberti C, Cohen Y: Mortality associated with late-onset pneumonia in the intensive care unit: results of a multi-center cohort study. *Intensive Care Med*. 2002;28:154-163

S98. Myny D, Depuydt P, Colardyn F, Blot S: Ventilator-associated pneumonia in a tertiary care ICU analysis of risk factors for acquisition and mortality. *Acta Clin Belg*. 2005;60:114-121.

S99. Nguile-Makao M, Zahar JR, Français A, Tabah A, Garrouste-Orgeas M, Allaouchiche B, Goldgran-Toledano D, Azoulay E, Adrie C, *JAMA*li S, Clec'h C. Attributable mortality of ventilator-associated pneumonia: respective impact of main characteristics at ICU admission and VAP onset using conditional logistic regression and multi-state models. *Intensive Care Med*.. 2010;36(5):781-9.

S100. Nielsen SL, Røder B, Magnussen P, Engquist A, Frimodt-møller N. Nosocomial pneumonia in an intensive care unit in a Danish university hospital: incidence, mortality and etiology. *Scand J Infect Dis*. 1992;24:65-70.

S101. Noor A, Hussain SF. Risk factors associated with development of ventilator associated pneumonia. *J Coll Physicians Surg Pak.* 2005;15:92–95.

S102. Nseir S, Di Pompeo C, Soubrier S, Cavestri B, Jozefowicz E, Saulnier F, Durocher A: Impact of ventilator-associated pneumonia on outcome in patients with COPD. *Chest*. 2005;128(3):1650-1656.

S103. [OUTCOMEREA] Saied WI, Mourvillier B, Cohen Y, Ruckly S, Reignier J, Marcotte G, et al on behalf of the OUTCOMEREA study group. A comparison of the mortality risk associated with ventilator-acquired bacterial pneumonia and nonventilator ICU-acquired bacterial pneumonia. *Crit Care Med*. 2019;47:345-52.

S104. Papazian L, Bregeon F, Thirion X, Gregoire R, Saux P, Denis JP, Perin G, Charrel J, Dumon JF, Affray JP, Gouin F: Effect of ventilator-associated pneumonia on mortality and morbidity. *Am J Respir Crit Care Med*. 1996;154:91-7.

S105. Pawar M, Mehta Y, Khurana P, Chaudhary A, Kulkarni V, Trehan N. Ventilator-associated pneumonia: incidence, risk factors, outcome, and microbiology. *J cardiothoracic & vasc anesthesia*. 2003 ;17(1):22-8.

S106. Plurad DS, Kim D, Bricker S, Lemesurier L, Neville A, Bongard F, Putnam B. Ventilator-associated pneumonia in severe traumatic brain injury: the clinical significance of admission *Chest* computed tomography findings. J surgical research. 2013;183(1):371-6.

S107. Pouwels KB, Vansteelandt S, Batra R, Edgeworth JD, Smieszek T, Robotham JV. Intensive care unit (ICU)-acquired bacteraemia and ICU mortality and discharge: addressing time-varying confounding using appropriate methodology. *J Hosp Infect.* 2018;99(1):42-7.

S108. Prowle JR, Echeverri JE, Ligabo EV, Sherry N, Taori GC, Crozier TM, Bellomo R. Acquired bloodstream infection in the intensive care unit: incidence and attributable mortality. *Crit Care* 2011;15(2):R100.

S109. Ramirez P, Lopez-Ferraz C, Gordon M, Gimeno A, Villarreal E, Ruiz J, Menendez R, Torres A. From starting mechanical ventilation to ventilator-associated pneumonia, choosing the right moment to start antibiotic treatment. *Crit Care* 2016;20(1):169.

S110. Rello J, Quintana E, Ausina V, Castella J, Luquin M, Net A, Prats G: Incidence, etiology, and outcome of nosocomial pneumonia in mechanically ventilated patients. *Chest*. 1991;100:439-444

S111. Rello J, Ricart M, Mirelis B, Quintana E, Gurgui M, Net A, Prats, G: Nosocomial bacteremia in a medical-surgical intensive care unit: epidemiologic characteristics and factors influencing mortality in 111 episodes. *Intensive Care Med* 1994;20:94-98.

S112. Rello J, Ollendorf DA, Oster G, et al. Epidemiology and outcomes of ventilator-associated pneumonia in a large US database. *Chest* 2002;122:2115-2121

S113. Rello J, Lorente C, Diaz E, et al. Incidence, etiology, and outcome of nosocomial pneumonia in ICU patients requiring percutaneous tracheotomy for mechanical ventilation. *Chest*. 2003;124:2239-2243.

S114. Resende MM, Monteiro SG, Callegari B, Figueiredo PM, Monteiro CR, Monteiro-Neto V. Epidemiology and outcomes of ventilator-associated pneumonia in northern Brazil: an analytical descriptive prospective cohort study. *BMC Infect Dis*. 2013;13(1):119.

S115. Reusser P, Zimmerli W, Scheidegger D, Marbet GA, Buser M, Gyr K: Role of gastric colonization in nosocomial infections and endotoxemia: a prospective study in neurosurgical patients on mechanical ventilation. *J Infect Dis*. 1989;160:414-421

S116. Rezai MS, Bagheri–Nesami M, Nikkhah A, Bayg AH. Incidence, risk factors, and outcome of ventilator-associated Pneumonia in 18 hospitals of Iran. Running title: ventilator-associated pneumonia in Iran. *Int J Adv Biotech Res.* 2016;7(3):936-46.

S117. Rincón-Ferrari MD, Flores-Cordero JM, Leal-Noval SR, Murillo-Cabezas F, Cayuelas A, Muñoz-Sánchez MA, Sánchez-Olmedo JI: Impact of ventilator-associated pneumonia in patients with severe head injury. J Trauma Acute Care Surg. 2004;57(6):1234-40.

S118. Rodrigues PM, Neto C, Santos LR, Knibel MF. Ventilator-associated pneumonia: epidemiology and impact on the clinical evolution of ICU patients. *J Brasileiro de Pneumologia.* 2009;35(11):1084-91.

S119. Rodriguez JL, Gibbons KJ, Bitzer LG, Dechert RE, Steinberg SM, Flint LM: Pneumonia: incidence, risk factors, and outcome in injured patients. *J Trauma*. 1991;31: 907-12.

S120. Rosenthal VD, Maki DG, Salomao R, Moreno CA, Mehta Y, Higuera F, Cuellar LE, Arikan ÖA, Abouqal R, Leblebicioglu H, International Nosocomial Infection Control Consortium*. Device-associated nosocomial infections in 55 intensive care units of 8 developing countries. *Annals Intern Med*. 2006;145(8):582-91.

S121. Rosenthal VD, Rodrigues C, Madani N, Mitrev Z, Ye G, Salomao R, Ulger F, Guanche-Garcell H, Kanj SS, Cuéllar LE, Higuera F. Effectiveness of a multidimensional approach for prevention of ventilator-associated pneumonia in adult intensive care units from 14 developing countries of four continents: findings of the International Nosocomial Infection Control Consortium. *Crit Care Med*. 2012;40(12):3121-8.

S122. Ruiz-Santana S, Garcia Jimenez A, Esteban A, et al. ICU pneumonias: a multi-institutional study. *Crit Care Med*. 1987;15:930-932.

S123. Salata RA, Lederman MM, Shlaes DM, Jacobs MR, Eckstein E, Tweardy D, Toossi Z, Chmielewski R, Marino J, King CH: Diagnosis of nosocomial pneumonia in intubated, intensive care unit patients. *Am Rev Respir Dis*. 1987;135:426-432

S124. Shahin J, Bielinski M, Guichon C, Flemming C, Kristof AS Suspected ventilator-associated respiratory infection in severely ill patients: a prospective observational study. *Crit Care* 2013;17(5): R251

S125. Sofianou DC, Constandinidis TC, Yannacou M, et al: Analysis of risk factors for ventilator-associated pneumonia in a multidisciplinary intensive care unit. *Eur J Clin Microbiol Infect Dis* 2000, 19:460-463.

S126. Stéphan F, Mabrouk N, Decailliot F, Delclaux C, Legrand P: Ventilator-associated pneumonia leading to acute lung injury after trauma: importance of *Haemophilus influenzae*. Anesthesiology. 2006;104: 235-41.

S127. Stoclin A, Rotolo F, Hicheri Y, Mons M, Chachaty E, Gachot B, Pignon JP, Wartelle M, Blot F. Ventilator-associated pneumonia and bloodstream infections in intensive care unit cancer patients: a retrospective 12-year study on 3388 prospectively monitored patients. Supportive Care in Cancer. 2020;28:193-200.

S128. Sutherland KR, Steinberg KP, Maunder RJ, Milberg JA, Allen DL, Hudson LD. Pulmonary infection during the acute respiratory distress syndrome. *Am J Respir Crit Care Med.*1995;152(2):550-6.

S129. Tan X, Zhu S, Yan D, Chen W, Chen R, Zou J, Yan J, Zhang X, Farmakiotis D, Mylonakis E. Candida spp. airway colonization: A potential risk factor for Acinetobacter baumannii ventilator-associated pneumonia. *Med Mycol*. 2016:myw009.

S130. Tejada Artigas AT, Dronda SB, Vallés EC, Marco JM, Usón MC, Figueras P, Suarez FJ, Hernandez A: Risk factors for nosocomial pneumonia in critically ill trauma patients. *Crit Care Med*. 2001;29:304-9.

S131. Thompson DS. Estimates of the rate of acquisition of bacteraemia and associated excess mortality in a general intensive care unit: a 10 year study. *J Hosp Infect*. 2008;69(1):56-61.

S132. Timsit JF, Chevret S, Valcke J, Misset B, Renaud B, Goldstein FW, Vaury P, Carlet J: Mortality of nosocomial pneumonia in ventilated patients: influence of diagnostic tools. *Am J Respir Crit Care Med.* 1996;154:116-23.

S133. Timsit JF, Schwebel C, Styfalova L, Cornet M, Poirier P, Forrestier C, Ruckly S, Jacob MC, Souweine B. Impact of bronchial colonization with Candida spp. on the risk of bacterial ventilator-associated pneumonia in the ICU: the FUNGIBACT prospective cohort study. *Intensive Care Med*. 2019;45:834-43.

S134. Torres A, Aznar R, Gatell JM, Jiménez P, González J, Ferrer A, et al: Incidence, risk, and prognosis factors of nosocomial pneumonia in mechanically ventilated patients. *Am Rev Respir Dis*. 1990;142:523-8.

S135. Trouillet JL, Chastre J, Vuagnat A, Joly-Guillou ML, Combaux D, et al: Ventilator-associated pneumonia caused by potentially drug-resistant bacteria. *Am J Respir Crit Care Med*. 1998;157(2):531-9.

S136. Urli T, Perone G, Acquarolo A, Zappa S, Antonini B, Ciani A: Surveillance of infections acquired in intensive care: usefulness in clinical practice. *J Hosp Infect* 2002, 52:130-5.

S137. Valles J, Pobo A, Garcia-Esquirol O, Mariscal D, Real J, Fernández R. Excess ICU mortality attributable to ventilator-associated pneumonia: the role of early vs late onset. *Intensive Care Med*, 2007;33(8):1363-1368.

S138. Vanhems P, Bénet T, Voirin N, Januel JM, Lepape A, Allaouchiche B, Argaud L, Chassard D, Guérin C. Early-onset ventilator-associated pneumonia incidence in intensive care units: a surveillance-based study. *BMC Infect Dis.* 2011;11(1):236.

S139. Verhamme KM, De Coster W, De Roo L, De Beenhouwer H, Nollet G, Verbeke J, Demeyer I, Jordens P: Pathogens in early-onset and late-onset intensive care unit–acquired pneumonia. *Infection Control Hospital Epidemiol.* 2007;28(4):389-397.

S140. Violan JS, Sanchez-Ramirez C, Mujica AP, Cendrero JC, Fernandez JA, de Castro FR: Impact of nosocomial pneumonia on the outcome of mechanically-ventilated patients. *Crit Care* (Lond). 1998;2:19-23.

S141. Warren DK, Zack JE, Elward AM, Cox MJ, Fraser VJ. Nosocomial primary bloodstream infections in intensive care unit patients in a nonteaching community medical center: a 21-month prospective study. *Clin Infect Dis.* 2001;33(8):1329-35.

S142. Woske HJ, Röding T, Schulz I, Lode H: Ventilator-associated pneumonia in a surgical intensive care unit Epidemiology, etiology and comparison of three bronchoscopic methods for microbiological specimen sampling. *Crit Care*. 2001;5:167–173.

S143. Xie DS, Xiong W, Lai RP, Liu L, Gan XM, Wang XH, Wang M, Lou YX, Fu XY, Wang HF, Xiang H. Ventilator-associated pneumonia in intensive care units in Hubei Province, China: a multicentre prospective cohort survey. *J Hosp Infect.* 2011;78(4):284-8.

S144. Zahar JR, Nguile-Makao M, Français A, Schwebel C, Garrouste-Orgeas M, Goldgran-Toledano D, Azoulay E, et al. Predicting the risk of documented ventilator-associated pneumonia for benchmarking: construction and validation of a score. *Crit Care Med*. 2009;37(9):2545-51.

S145. Apisarnthanarak A, Pinitchai U, Thongphubeth K, Yuekyen C, Warren DK, Zack JE, Warachan B, Fraser VJ. Effectiveness of an educational program to reduce ventilator-associated pneumonia in a tertiary care center in Thailand: a 4-year study. *Clin Infect Dis*. 2007;45(6):704-11.

S146. François B, Jafri HS, Chastre J, Sánchez-García M, Eggimann P, Dequin PF, Huberlant V, Soria LV, Boulain T, Bretonnière C, Pugin J. Efficacy and safety of suvratoxumab for prevention of Staphylococcus aureus ventilator-associated pneumonia (SAATELLITE): a multicentre, randomised, double-blind, placebo-controlled, parallel-group, phase 2 pilot trial. *Lancet* Infect Dis. 2021;21(9):1313-23.

S147. Acosta-Escribano J, Fernández-Vivas M, Carmona TG, Caturla-Such J, Garcia-Martinez M, Menendez-Mainer A, Sanchez-Payá J (2010) Gastric versus transpyloric feeding in severe traumatic brain injury: a prospective, randomized trial. *Intensive Care Med* 36:1532-1539

S148. Bonten MJ, Gaillard CA, Van der Geest S, Van Tiel FH, Beysens AJ, Smeets HG, Stobberingh EE: The role of intragastric acidity and stress ulcer prophylaxis on colonization and infection in mechanically ventilated ICU patients. A stratified, randomized, double-blind study of sucralfate versus antacids. *Am J Respir Crit Care Med*. 1995;152:1825-1834.

S149. Cook D, Guyatt G, Marshall J, et al A comparison of sucralfate and ranitidine for the prevention of upper gastrointestinal bleeding in patients requiring mechanical ventilation. Canadian Critical Care Trials Group. *N Engl J Med* 1998;338:791-797

S150. Driks MR, Craven DE, Celli BR, et al Nosocomial pneumonia in intubated patients given sucralfate as compared with antacids or histamine type 2 blockers. The role of gastric colonization. *N Engl J Med* 1987; 317:1376-1382

S151. Forestier C, Guelon D, Cluytens V, Guillart T, Sirot J, De champs C: Oral probiotic and prevention of *Pseudomonas aeruginosa* infections: a randomized, double-blind, placebocontrolled pilot study in intensive care unit patients. *Crit Care* 2008;12:R69.

S152. Giamarellos-Bourboulis EJ, Bengmark S, Kanellakopoulou K, Kotzampassi K. Pro-and synbiotics to control inflammation and infection in patients with multiple injuries. J Trauma and Acute Care Surgery. 2009;67(4):815-21.

S153. Heyland DK, Cook DJ, Schoenfeld PS, Frietag A, Varon J, Wood G The effect of acidified enteral feeds on gastric colonization in critically ill patients: results of a multicenter randomized trial. Canadian Critical Care Trials Group. *Crit Care Med* 1999;27:2399-2406

S154. Knight DJ, Gardiner D, Banks A, Snape SE, Weston VC, Bengmark S, Girling KJ: Effect of synbiotic therapy on the incidence of ventilator associated pneumonia in critically ill patients: a randomised, double-blind, placebo-controlled trial. *Intensive Care Med*. 2009;35:854-861.

S155. Kostadima E, Kaditis AG, Alexopoulos EI, Zakynthinos E, Sfyras D. Early gastrostomy reduces the rate of ventilator-associated pneumonia in stroke or head injury patients. Eur Respir J. 2005;26(1):106-11.

S156. Laggner AN, Lenz K, Base W, Druml W, Schneeweiss B, Grimm G. Prevention of upper gastrointestinal bleeding in long-term ventilated patients: Sucralfate versus ranitidine. Am J Med. 1989;86(6):81-4.

S157. Martin C, Perrin G, Gevaudan MJ, Saux P, Gouin F. Heat and moisture exchangers and vaporizing humidifiers in the intensive care unit. *Chest*. 1990;97(1):144-9.

S158. Miano TA, Reichert MG, Houle TT, MacGregor DA, Kincaid EH, Bowton DL. Nosocomial pneumonia risk and stress ulcer prophylaxis: a comparison of pantoprazole vs ranitidine in cardiothoracic surgery patients. *Chest*. 2009;136(2):440-7.

S159. Montecalvo MA, Steger KA, Farber HW, Smith BF, Dennis RC, Fitzpatrick GF, Pollack SD, Korsberg TZ, Birkett DH, Hirsch EF. Nutritional outcome and pneumonia in critical care patients randomized to gastric versus jejunal tube feedings. The Critical Care Research Team. *Crit Care Med*.. 1992 ;20(10):1377-87.

S160. Morrow LE, Kollef MH, Casale TB: Probiotic prophylaxis of ventilator-associated pneumonia: a blinded, randomized, controlled trial. *Am J Respir Crit Care Med*. 2010;182:1058-1064

S161. Pickworth KK, Falcone RE, Hoogeboom JE, et al Occurrence of nosocomial pneumonia in mechanically ventilated trauma patients: a comparison of sucralfate and ranitidine. *Crit Care Med* 1993;21:1856-1862

S162. Prod'hom G, Leuenberger P, Koerfer J, Blum A, Chiolero R, Schaller MD, Perret C, Spinnler O, Blondel J, Siegrist H, Saghafi L: Nosocomial pneumonia in mechanically ventilated patients receiving antacid, ranitidine, or sucralfate as prophylaxis for stress ulcer. A randomized controlled trial. Ann Intern Med. 1994;120:653-62.

S163. Reignier J, Mercier E, Le Gouge A, Boulain T, Desachy A, Bellec F, Lascarrou JB: Effect of Not Monitoring Residual Gastric Volume on Risk of Ventilator-Associated Pneumonia in Adults Receiving Mechanical Ventilation and Early Enteral Feeding. A Randomized Controlled Trial. *JAMA* 2013, 309;249-256.

S164. Ryan P, Dawson J, Teres D, Celoria G, Navab F: Nosocomial pneumonia during stress ulcer prophylaxis with cimetidine and sucralfate. Arch Surg. 1993;128(12):1353-7.

S165. Zeng J, Wang CT, Zhang FS, Qi F, Wang SF, Ma S, Wu TJ, Tian H, Tian ZT, Zhang SL, Qu Y. Effect of probiotics on the incidence of ventilator-associated pneumonia in critically ill patients: a randomized controlled multicenter trial. Intens care med. 2016;42(6):1018-28.

S166. Damas P, Legrain C, Lambermont B, Dardenne N, Guntz J, Kisoka G, Demaret P, Rousseau AF, Jadot L, Piret S, Noirot D. Prevention of ventilator-associated pneumonia by noble metal coating of endotracheal tubes: a multi-center, randomized, double-blind study. *Annals of intensive care*. 2022;12:1-0.

S167. Dat VQ, Minh Yen L, Thi Loan H, Dinh Phu V, Thien Binh N, Geskus RB, Khanh Trinh DH, et al. Effectiveness of continuous endotracheal cuff pressure control for the prevention of ventilator-associated respiratory infections: An open-label randomized, controlled trial. *Clin Infect Dis*. 2022;74(10):1795-803.

S168. Djedaini K, Billiard M, Mier L, Le Bourdelles G, Brun P, et al: Changing heat and moisture exchangers every 48 hours rather than 24 hours does not affect their efficacy and the incidence of nosocomial pneumonia. *Am J Respir Crit Care Med*. 1995;152(5):1562-9.

S169. Drakulovic MB, Torres A, Bauer TT, et al: Supine body position as a risk factor for nosocomial pneumonia in mechanically ventilated patients: a randomised trial. *Lancet*. 1999;354(9193):1851-1858

S170. Dreyfuss D, Djedaini K, Weber P, Brun P, Lanore JJ, Rahmani J, Coste F: Prospective study of nosocomial pneumonia and of patient and circuit colonization during mechanical ventilation with circuit changes every 48 hours versus no change. *Am Rev Respir Dis*. 1991;143(4 Pt 1), 738-743.

S171. Dreyfuss D, Djedaïni K, Gros I, Mier L, Le Bourdellés G, Cohen Y, Estagnasié P, Coste F, Boussougant Y: Mechanical ventilation with heated humidifiers or heat and moisture exchangers: effects on patient colonization and incidence of nosocomial pneumonia. *Am J Respir Crit Care Med.* 1995;151:986-92.

S172. Holzapfel L, Chastang C, Demingeon G, Bohe J, Piralla B, Coupry A: A randomized study assessing the systematic search for maxillary sinusitis in nasotracheally mechanically ventilated patients. Influence of nosocomial maxillary sinusitis on the occurrence of ventilator-associated pneumonia. *Am J Respir Crit Care Med.* 1999;159:695-701

S173. Kirton OC, DeHaven B, Morgan J, et al. A prospective, randomized comparison of an in-line heat moisture exchange filter and heated wire humidifiers: rates of ventilator-associated early-onset (community-acquired) or late-onset (hospital-acquired) pneumonia and incidence of endotracheal tube occlusion. *Chest* 1997;112:1055-9.

S174. Kollef MH, Vlasnik JO, Sharpless L, Pasque C, Murphy D, Fraser V. Scheduled change of antibiotic classes: a strategy to decrease the incidence of ventilator-associated pneumonia. *Am J Respir Crit Care Med.* 1997;156(4):1040-8.

S175. Kollef MH, Afessa B, Anzueto A, Veremakis C, Kerr KM, Margolis BD, Schinner R: Silver-coated endotracheal tubes and incidence of ventilator-associated pneumonia: the NASCENT randomized trial. *JAMA*. 2008;300(7):805-813

S176. Lacherade JC, Auburtin M, Cerf C, Van de Louw A, Soufir L, Rebufat Y, Rezaiguia S, Ricard JD, Lellouche F, Brun-Buisson C, Brochard L: Impact of humidification systems on ventilator-associated pneumonia: a randomized multicenter trial. *Am J Respir Crit Care Med*. 2005;172:1276-1282

S177. Lacherade JC, De Jonghe B, Guezennec P, Debbat K, Hayon J, Monsel A, Bastuji-Garin S: Intermittent subglottic secretion drainage and ventilator-associated pneumonia A multicenter trial. *Am J Respir Crit Care Med*. 2010;182:910-917.

S178. Launey Y, Nesseler N, Le Cousin A, Feuillet F, Garlantezec R, et al: Effect of a fever control protocol-based strategy on ventilator-associated pneumonia in severely brain-injured patients. *Crit Care*. 2014;18(6):1.

S179. Lorente L, Lecuona M, Málaga J, Revert C, Mora ML, Sierra A: Bacterial filters in respiratory circuits: an unnecessary cost? *Crit Care Med* 2003;31:2126-2130

S180. Lorente L, Lecuona M, Galván R, Ramos MJ, Mora ML, Sierra A: Periodically changing ventilator circuits is not necessary to prevent ventilator-associated pneumonia when a heat and moisture exchanger is used. *Infect Control Hosp Epidemiol*. 2004;25:1077-1082

S181. Lorente L, Lecuona M, Martín MM, García C, Mora ML, Sierra A: Ventilator-associated pneumonia using a closed versus an open tracheal suction system. *Crit Care Med*. 2005;33:115-119

S182. Lorente L, Lecuona M, Jiménez A, Mora ML, Sierra A: Tracheal suction by closed system without daily change versus open system. *Intensive Care Med*. 2006;32:538-44.

S183. Lorente L, Lecuona M, Jimenez A, Mora ML, Sierra A: Ventilator-associated pneumonia using a heated humidifier or a heat and moisture exchanger: a randomized controlled trial. *Crit Care* 2006;10:R116

S184. Lorente L, Lecuona M, Jimenez A, Mora ML, Sierra: Influence of an endotracheal tube with polyurethane cuff and subglottic secretion drainage on pneumonia. *Am J Respir Crit Care Med*. 2007;176:1079-1083

S185. Lorente L, Lecuona M, Jiménez A, Lorenzo L, Roca I, Cabrera J, et al: Continuous endotracheal tube cuff pressure control system protects against ventilator-associated pneumonia. *Crit Care*. 2014;18(2):1.

S186. Mahmoodpoor A, Hamishehkar H, Hamidi M, Shadvar K, Sanaie S, Golzari SE, Khan ZH, Nader ND. A prospective randomized trial of tapered-cuff endotracheal tubes with intermittent subglottic suctioning in preventing ventilator-associated pneumonia in critically ill patients. *J Critical Care.* 2017;38:152-6.

S187. Manzano F, Fernandez-Mondejar E, Colmenero M, Poyatos ME, Rivera R, Machado J, Catalan I, Artigas A: Positive-end expiratory pressure reduces incidence of ventilator-associated pneumonia in nonhypoxemic patients. *Crit Care Med*: 2008;36(8):2225-31.

S188. Marjanovic N, Boisson M, Asehnoune K, Foucrier A, Lasocki S, Ichai C, Leone M, Pottecher J, Lefrant JY, Falcon D, Veber B. Continuous pneumatic regulation of tracheal cuff pressure to decrease ventilator-associated pneumonia in trauma patients who were mechanically ventilated: the AGATE multicenter randomized controlled study. *Chest*. 2021;160(2):499-508.

S189. Nseir S, Zerimech F, Fournier C, Lubret R, Ramon P, et al: Continuous control of tracheal cuff pressure and microaspiration of gastric contents in critically ill patients. *Am J Respir Crit Care Med*. 2011;184(9):1041-7.

S190. Pneumatikos I, Konstantonis D, Tsagaris I, Theodorou V, Vretzakis G, Danielides V, Bouros D: Prevention of nosocomial maxillary sinusitis in the ICU: the effects of topically applied alpha-adrenergic agonists and corticosteroids. *Intensive Care Med*. 2006;32:532-537

S191. Rumbak MJ, Truncale T, Newton MN, Adams B, Hazard P. A Prospective, Randomized Study Comparing Early Versus Delayed Percutaneous Tracheostomy In Critically Ill Medical Patients Requiring Prolonged Mechanical Ventilation. *Chest*. 2000;118(4):97S-8S.

S192. Smulders K, van der Hoeven H, Weers-Pothoff I, Vandenbroucke-Grauls C A randomized clinical trial of intermittent subglottic secretion drainage in patients receiving mechanical ventilation. *Chest* 2002;121:858-862

S193. Staudinger T, Bojic A, Holzinger U, Meyer B, Rohwer M, Mallner F, Locker GJ Continuous lateral rotation therapy to prevent ventilator-associated pneumonia *Crit Care Med* 2010;38(2):486-490

S194. Thomachot L, Viviand X, Arnaud S, Boisson C, Martin CD: Comparing two heat and moisture exchangers, one hydrophobic and one hygroscopic, on humidifying efficacy and the rate of nosocomial pneumonia. *Chest*. 1998;114:1383-1389

S195. Thomachot L, Leone M, Razzouk K, Antonini F, Vialet R, Martin C: Do the components of heat and moisture exchanger filters affect humidifying efficacy and the incidence of nosocomial pneumonia? *Crit Care Med*. 1999;27:923–928

S196. Thomachot L, Leone M, Razzouk K, Antonini F, Vialet R, Martin C: Randomized Clinical Trial of Extended Use of a Hydrophobic Condenser Humidifier: 1 vs 7 Days. *Crit Care Med*. 2002;30:232-7

S197. Valencia M, Ferrer M, Farre R, Navajas D, Badia JR, et al: Automatic control of tracheal tube cuff pressure in ventilated patients in semirecumbent position: a randomized trial. *Crit Care Med*. 2007;35: 1543-9.

S198. Walaszek M, Gniadek A, Kolpa M, Wolak Z, Kosiarska A. The effect of subglottic secretion drainage on the incidence of ventilator associated pneumonia. Biomed Pap Med Fac Univ Palacky Olomouc Czech Repub. 2017;161(4):374-80.

S199. Bleasdale SC, Trick WE, Gonzalez IM, Lyles RD, Hayden MK, Weinstein RA. Effectiveness of chlorhexidine bathing to reduce catheter-associated bloodstream infections in medical intensive care unit patients. Arch intern med. 2007;167(19):2073-9.

S200. Ćabov T, Macan D, Husedžinović I, Škrlin-Šubić J, Bošnjak D, Šestan-Crnek S, Perić B, Kovač Z, Golubović V. The impact of oral health and 0.2% chlorhexidine oral gel on the prevalence of nosocomial infections in surgical intensive-care patients: a randomized placebo-controlled study. Wiener klinische Wochenschrift. 2010;122(13):397-404.

S201. Caruso P, Denari S, Ruiz SA, Demarzo SE, Deheinzelin D Saline instillation before tracheal suctioning decreases the incidence of ventilator-associated pneumonia. *Crit Care Med* 2009;37:32-38

S202. Climo MW, Yokoe DS, Warren DK, Perl TM, Bolon M, Herwaldt LA, Weinstein RA, Sepkowitz KA, Jernigan JA, Sanogo K, Wong ES. 2013. Effect of daily chlorhexidine bathing on hospital-acquired infection. *N Engl J Med* 368:533–542

S203. Fourrier FE, Cau-Pottier H, Boutigny M, Roussel-Delvallez M, Jourdain, Chopin C: Effects of dental plaque antiseptic decontamination on bacterial colonization and nosocomial infections in critically ill patients. *Intensive Care Med*. 2000;26:1239-1247

S204. Fourrier F, Dubois D, Pronnier P, Herbecq P, Leroy O, Desmettre T, Roussel-Delvallez M: Effect of gingival and dental plaque antiseptic decontamination on nosocomial infections acquired in the intensive care unit a double-blind placebo-controlled multicenter study. *Crit Care Med*. 2005;33:1728-1735

S205. Gacouin A, Barbarot N, Camus C, Salomon S, Isslame S, Marque S, Lavoué S et al Late-onset ventilator-associated pneumonia in nontrauma intensive care unit patients. Anesthesia & Analgesia. 2009;109(5):1584-90.

S206. Genuit T, Bochicchio G, Napolitano LM, McCarter RJ, Roghman MC. Prophylactic chlorhexidine oral rinse decreases ventilator-associated pneumonia in surgical ICU patients. Surg Infect (Larchmt). 2001;2:5-18.

S207. Huang SS, Septimus E, Kleinman K, Moody J, Hickok J, Avery TR, Lankiewicz J, et al 2013. Targeted versus universal de- colonization to prevent ICU infection. *N Engl J Med* 368:2255–2265

S208. Koeman M, van der Ven AJ, Hak E, et al. Oral decontamination with chlorhexidine reduces the incidence of ventilator-associated pneumonia. *Am J Respir Crit Care Med* 2006;173:1348-1355

S209. Kollef M, Pittet D, Sanchez Garcia M, et al. A randomized double-blind trial of iseganan in prevention of ventilator-associated pneumonia. *Am J Respir Crit Care Med* 2006: 173:91-7.

S210. Lorente L, Lecuona M, Jiménez A, Palmero S, Pastor E, Lafuente N, Ramos MJ, Mora ML, Sierra A: Ventilator-associated pneumonia with or without toothbrushing a randomized controlled trial. Eur J Clin Microbiol Infect Dis. 2012;31:1-9

S211. Milstone AM, Elward A, Song X, Zerr DM, Orscheln R, Speck K, Obeng D, Reich NG, Coffin SE, Perl TM. 2013. Daily chlorhexidine bathing to reduce bacteraemia in critically ill children: a multicentre, cluster-randomised, crossover trial. *Lancet* 381:1099 –1106

S212. Mori H, Hirasawa H, Oda S, Shiga H, Matsuda K, Nakamura M Oral care reduces incidence of ventilator-associated pneumonia in ICU populations. *Intensive Care Med*. 2006;32(2):230-236

S213. Morris AC, Hay AW, Swann DG, Everingham K, McCulloch C, McNulty J, Brooks O, Laurenson IF, Cook B, Walsh TS. Reducing ventilator-associated pneumonia in intensive care: impact of implementing a care bundle. *Crit Care Med*. 2011;39(10):2218-24.

S214. Noto MJ, Domenico HJ, Byrne DW, Talbot T, Rice TW, Bernard GR, Wheeler AP. 2015. Chlorhexidine bathing and health care-associated infections: a randomized clinical trial. *JAMA* 313:369 –378.

S215. Paling FP, Wolkewitz M, Bode LG, Klouwenberg PK, Ong DS, Depuydt P, de Bus L, Sifakis F, Bonten MJ, Kluytmans JA. *Staphylococcus aureus* colonization at ICU admission as a risk factor for developing *S. aureus* ICU pneumonia. *Clin Microbiol Infect*. 2017;23(1):49-e9.

S216. Pobo A, Lisboa T, Rodriguez A, Sole R, Magret M, Trefler S, Gómez F, Rello J. A randomized trial of dental brushing for preventing ventilator-associated pneumonia. *Chest* 2009;136:433-439.

S217. Roquilly A, Mahe PJ, Seguin P, Guitton C, Floch H, Tellier AC, Merson L, Renard B, Malledant Y, Flet L, Sebille V. Hydrocortisone therapy for patients with multiple trauma: the randomized controlled HYPOLYTE study. *JAMA*. 2011;305(12):1201-9.

S218. Sebastian MR, Lodha R, Kapil A, Kabra SK. Oral mucosal decontamination with chlorhexidine for the prevention of ventilator-associated pneumonia in children—a randomized, controlled trial. *Pediatric Crit Care Med*. 2012;13(5):e305-10.

S219. Segers P, Speekenbrink RG, Ubbink DT, van Ogtrop ML, de Mol BA. Prevention of nosocomial infection in cardiac surgery by decontamination of the nasopharynx and oropharynx with chlorhexidine gluconate: a randomized controlled trial. *JAMA*. 2006;296(20):2460-6.

S220. Seguin P, Tanguy M , Laviolle B, Tirel O, et al: Effect of oropharyngeal decontamination by povidone-iodine on ventilator-associated pneumonia in patients with head trauma. *Crit Care Med* 2006, 34:1514-1519.

S221. Seguin P, Laviolle B, Dahyot-Fizelier C, Dumont R, Veber B, Gergaud S, Asehnoune K, et al. Effect of oropharyngeal povidone-iodine preventive oral care on ventilator-associated pneumonia in severely brain-injured or cerebral hemorrhage patients: a multicenter, randomized controlled trial. *Crit Care Med*. 2014;42(1):1-8.

S222. Beovic B, Matos B, Bošnjak R, Seme K, Mueller-Premru M, Hergouth-Krizan V, Cizman M. Prevention of nosocomial lower respiratory tract infections in patients after intracranial artery aneurysm surgery with a short course of antimicrobials. *Internat J Antimicrob Agents*. 2003;22(1):60-6.

S223. Bergmans DC, Bonten MJ, Gaillard CA, et al Prevention of ventilator-associated pneumonia by oral decontamination: a prospective, randomized, double-blind, placebo-controlled study. *Am J Respir Crit Care Med* 2001;164:382-388

S224. Biagioni E, Ferrari E, Gatto I, Serio L, Farinelli C, Coloretti I, Talamonti M, Tosi M, Meschiari M, Tonelli R, Venturelli C. Role of Selective Digestive Decontamination in the Prevention of Ventilator-Associated Pneumonia in COVID-19 Patients: A Pre-Post Observational Study. *J Clinical Medicine*. 2023;12(4):1432.

S225. Bonten MJ, Gaillard CA, Johanson Jr WG, Van Tiel FH, Smeets HG, Van Der Geest S, Stobberingh EE. Colonization in patients receiving and not receiving topical antimicrobial prophylaxis. *Am J Respir Crit Care Med* 1994;150(5):1332-1340.

S226. Bos LD, Stips C, Schouten LR, van Vught LA, Wiewel MA, Wieske L, van Hooijdonk RT, Straat M, de Beer FM, Glas GJ, Visser CE. Selective decontamination of the digestive tract halves the prevalence of ventilator-associated pneumonia compared to selective oral decontamination. *Intensive Care Med*. 2017;43:1535-7.

S227. Camus C, Salomon S, Bouchigny C, Gacouin A, Lavoué S, Donnio PY, Bellissant E (2014) Short-Term Decline in All-Cause Acquired Infections With the Routine Use of a Decontamination Regimen Combining Topical Polymyxin, Tobramycin, and Amphotericin B With Mupirocin and Chlorhexidine in the ICU: A Single-Center Experience. *Crit Care Med* 42:1121-1130

S228. David A. Infektionen und Keimspektrum nach einer Dekade der Selektiven Darmdekontamination (SDD) in der operativen Intensivmedizin (Doctoral dissertation, Münster (Westfalen), Univ., Diss., 2007).

S229. De la court Jara R, Sigaloff KC, Groot T, van der Spoel JI, Schade RP. Reducing the dosing frequency of selective digestive tract decontamination to three times daily provides effective decontamination of Gram-negative bacteria. *Eur J Clin Microbiol Infect Dis*. 2021 1:1-8.

S230. de Smet AMGA, Kluytmans JAJW, Cooper BS, et al: Decontamination of the digestive tract and oropharynx in ICU patients. *N Engl J Med* 2009, 360:20–31.

S231. Garbino J, Lew DP, Romand JA, Hugonnet S, Auckenthaler R, Pittet D. Prevention of severe Candida infections in nonneutropenic, high-risk, critically ill patients: a randomized, double-blind, placebo-controlled trial in patients treated by selective digestive decontamination. *Intensive Care Med*. 2002;28:1708-17.

S232. Hartenauer UB, Thülig B, Lawin P, Fegeler W. Infection surveillance and selective decontamination of the digestive tract (SDD) in critically ill patients—results of a controlled study. *Infection.* 1990;18(1):S22-30.

S233. Hjortrup A, Rasmussen A, Hansen BA, Hoiby N, Heslet L, et al (1997) Early bacterial and fungal infections in liver transplantation after oral selective bowel decontamination. *Transpl proc* 29:3106-3110

S234. Landelle C, Boyer VN, Abbas M, Genevois E, Abidi N, Naimo S, Raulais R, Bouchoud L, Boroli F, Terrisse H, Bosson JL. Impact of a multifaceted prevention program on ventilator-associated pneumonia including selective oropharyngeal decontamination. *Intensive Care Med* 2018;44:1777-86.

S235. Leone M, Bourgoin A, et al. Influence on outcome of ventilator-associated pneumonia in multiple trauma patients with head trauma treated with selected digestive decontamination. *Crit Care Med* 2002; 30:1741-6.

S236. Massart N, Reizine F, Dupin C, Legay F, Legris E, Cady A, Rieul G, Barbarot N, Magahlaes E, Fillatre P. Prevention of acquired invasive fungal infection with decontamination regimen in mechanically ventilated ICU patients: a pre/post observational study. *Infect Dis*. 2023;55(4):263-71.

S237. Nardi G, Valentinis U, Bartaletti R, Bello A, De AM, Muzzi R, Giordano F, Troncon MG. Effectiveness of topical selective decontamination, without systemic antibiotic prophylaxis, in prevention of pulmonary infection in intensive care. *Minerva anestesiologica.* 1990;56(1-2):19-26.

S238. Nardi G, Di Silvestre A, De Monte A, Massarutti D, Proietti A, Troncon MG, Zussino M: Reduction in gram-positive pneumonia and antibiotic consumption following the use of a SDD protocol including nasal and oral mupirocin. Eur J Emerg Med 2001;8:203-214

S239. Ong DS, Bonten MJ, Safdari K, Spitoni C, Frencken JF, Witteveen E, Horn J, Klein Klouwenberg PM, Cremer OL, MARS consortium, de Beer FM. Epidemiology, management, and risk-adjusted mortality of ICU-acquired enterococcal bacteremia. *Clin Infect Dis* 2015;61(9):1413-20.

S240. Oostdijk EAN, Kesecioglu J, Schultz MJ, et al. Notice of Retraction and Replacement: Oostdijk et al. Effects of Decontamination of the Oropharynx and Intestinal Tract on Antibiotic Resistance in ICUs: A Randomized Clinical Trial. *JAMA*. 2014;312(14):1429-1437. *JAMA* 2017

S241. Rouby JJ, Poete P, de Lassale EM, Nicolas MH, Bodin L, et al. Prevention of Gram negative noscomial bronchopneumonia by intratracheal colistin in critically ill patients. *Intensive Care Med*. 1994;20(3):187-92.

S242. Silvestri L, Bragadin CM, Milanese M, Gregori D, Consales C, Gullo A, Van Saene HK. Are most ICU infections really nosocomial? A prospective observational cohort study in mechanically ventilated patients. *J Hosp Infect.* 1999;42(2):125-33.

S243. Silvestri L, Milanese M, Oblach L, Fontana F, Gregori D, Guerra R, van Saene HK. Enteral vancomycin to control methicillin-resistant Staphylococcus aureus outbreak in mechanically ventilated patients. Amer J infect control. 2002 ;30(7):391-9.

S244. Silvestri L, Van Saene HK, Milanese M, Fontana F, Gregori D, Oblach L, et al. Prevention of MRSA pneumonia by oral vancomycin decontamination: a randomised trial. Eur Respir J. 2004;23(6):921-6.

S245. Steffen R, Reinhartz O, Blumhardt G, Bechstein WO, Raakow R, Langrehr JM, Rossaint R, Slama K, Neuhaus P. Bacterial and fungal colonization and infections using oral selective bowel decontamination in orthotopic liver transplantations. *Transpl Inter.* 1994;7(2):101-8.

S246. Stoutenbeek CP, van Saene HK, Miranda DR, Zandstra DF, Langrehr D; The effect of oropharyngeal decontamination using topical nonabsorbable antibiotics on the incidence of nosocomial respiratory tract infections in multiple trauma patients. J Trauma 1987;27:357-364

S247. The SuDDICU Investigators for the Australian and New Zealand Intensive Care Society Clinical Trials Group. Effect of selective decontamination of the digestive tract on hospital mortality in critically ill patients receiving mechanical ventilation: a randomized clinical trial. *JAMA*. 2022. doi:10.1001/*JAMA*.2022.17927

S248. Veelo DP, Bulut T, Dongelmans DA, et al. The incidence and microbial spectrum of ventilator-associated pneumonia after tracheotomy in a selective decontamination of the digestive tract-setting. *J Infect* 2008; 56:20-6.

S249. Winter R, Humphreys H, Pick A, et al: A controlled trial of selective decontamination of the digestive tract in intensive care and its effect on nosocomial infection. *J Antimicrob Chemother*. 1992;30:73-87

S250. Wittekamp BH, Plantinga NL, Cooper BS, Lopez-Contreras J, Coll P, Mancebo J, Wise MP, Morgan MP, Depuydt P, Boelens J, Dugernier T. Decontamination strategies and bloodstream infections with antibiotic-resistant microorganisms in ventilated patients: a randomized clinical trial. *JAMA*. 2018 ;320(20):2087-98.

S251. Abele-Horn M, Dauber A, Bauernfeind A, Russwurm W, Seyfarth-Metzger I, Gleich P, Ruckdeschel G: Decrease in nosocomial pneumonia in ventilated patients by selective oropharyngeal decontamination (SOD). *Intensive Care Med*. 1997;23:187-95.

S252. Aerdts SJ, van Dalen R, Clasener HA, Festen J, van Lier HJ, Vollaard EJ: Antibiotic prophylaxis of respiratory tract infection in mechanically ventilated patients. A prospective, blinded, randomized trial of the effect of a novel regimen. *Chest*. 1991;100:783-791

S253. Blair P, Rowlands BJ, Lowry K, Webb H, Armstrong P, et al. Selective decontamination of the digestive tract: a stratified, randomized, prospective study in a mixed intensive care unit. Surgery 1991;110:303-309

S254. Camus C, Bellissant E, Sebille V, Perrotin D, Garo B, Legras A, Renault A, Le Corre P, Donnio PY, Gacouin A: Prevention of acquired infections in intubated patients with the combination of two decontamination regimens. *Crit Care Med* 2005, 33:307-314.

S255. Cerra FB, Maddaus MA, Dunn DL, Wells CL, Konstantinides NN, Lehmann SL, Mann HJ. Selective gut decontamination reduces nosocomial infections and length of stay but not mortality or organ failure in surgical intensive care unit patients. *Arch Surg* 1992;127:163-167.

S256. de La Cal MA, Cerdá E, Garcıa-Hierro P, Van Saene HK. G ómez-Santos D, Negro E & Lorente JA. Survival benefit in critically ill burned patients receiving selective decontamination of the digestive tract: a randomized, placebocontrolled, double-blind trial. *Ann Surg*. 2005;241:424-30.

S257. de Latorre FJ, Pont T, Ferrer A, Rosselló J, Palomar M, Planas M. Pattern of tracheal colonization during mechanical ventilation. *Am J Respir Crit Care Med*. 1995;152(3):1028-33.

S258. Ferrer M, Torres A, Gonzalez J, Puig de la Bellacasa J, el-Ebiary M, Roca M, Gatell JM, et al: Utility of selective digestive decontamination in mechanically ventilated patients. *Ann Intern Med.* 1994;120:389-395

S259. Gastinne HM, Wolff M, Delatour F, Faurisson F, Chevret S. A controlled trial in intensive care units of selective decontamination of the digestive tract with nonabsorbable antibiotics. *New Engl J Med.* 1992;326(9):594-9.

S260. Gaussorgues P, Salord M, Sirodot S, Tigaud S, Cagnin S, Gerard M, Robert D. Efficiency of selective decontamination of the digestive tract on the occurrence of nosocomial bacteremia in patients on mechanical ventilation receiving betamimetic therapy. Réan Soins Intens Méd Urg 1991;7:169-174.

S261. Georges B, Mazerolles M, Decun J-F, et al. Décontamination digestive sélective résultats d'une étude chez le polytraumatisé. *Réanimation Soins Intensifs Médecin d'Urgence* 1994; 3: 621-7.

S262. Hammond JM, Potgieter PD, Saunders LG. Selective decontamination of the digestive tract in multiple trauma patients-Is there a role? Results of a prospective, double-blind, randomized trial. *Crit Care Med*. 1994;22(1):33-9.

S263. Jacobs S, Foweraker JE, Roberts SE: Effectiveness of selective decontamination of the digestive tract (SDD) in an ICU with a policy encouraging a low gastric pH. *Clin Intensive Med*. 1992;3:52-58

S264. Karvouniaris M, Makris D, Zygoulis P, Triantaris A, Xitsas S, Mantzarlis K, Petinaki E, Zakynthinos E. Nebulised colistin for ventilator-associated pneumonia prevention. *Eur Resp J.* 2015:46:1544-1547.

S265. Korinek AM, Laisne MJ, Nicolas MH, Raskine L, Deroin V, Sanson-lepors MJ: Selective decontamination of the digestive tract in neurosurgical intensive care unit patients: a double-blind, randomized, placebo-controlled study. *Crit Care Med*. 1993;21:1466-73.

S266. Laggner AN, Tryba M, Georgopoulos A, Lenz K, Grimm G, Graninger W, Schneeweiss B, Druml W (1994) Oropharyngeal decontamination with gentamicin for long-term ventilated patients on stress ulcer prophylaxis with sucralfate? Wien Klin Wochenschr 106:15-19

S267. Langlois-Karaga A, Bues-Charbit M, Davignon A, Albanese J, Durbec O, Martin C, Morati N, Balansard G. Selective digestive decontamination in multiple trauma patients: cost and efficacy. Pharmacy World & Science. 1995 ;17(1):12-6.

S268. Palomar M, Alvarez-Lerma F, Jorda R, Bermejo B, Catalan Study Group of Nosocomial Pneumonia Prevention: Prevention of nosocomial infection in mechanically ventilated patients: selective digestive decontamination versus sucralfate. Clin Intens Care. 1997;8:228-235

S269. Quinio B, Albanese J, Bues-Charbit M, Viviand X, Martin C; Selective decontamination of the digestive tract in multiple trauma patients. A prospective double-blind, randomized, placebo-controlled study. *Chest* 1996;109:765-772

S270. Reizine F, Asehnoune K, Roquilly A, Laviolle B, Rousseau C, Arnouat M, Dahyot-Fizelier C, Seguin P. Effects of antibiotic prophylaxis on ventilator-associated pneumonia in severe traumatic brain injury. A post hoc analysis of two trials. *J Critical Care*. 2019;50:221-6.

S271. Rimola A, Bory F, Teres J, Perez-Ayuso RM, Arroyo V, Rodes J. Oral, nonabsorbable antibiotics prevent infection in cirrhotics with gastrointestinal hemorrhage. *Hepatol*. 1985;5(3):463-7.

S272. Rocha LA, Martin MJ, Pita S, Paz J, Seco C, Margusino L, Villanueva R, Duran MT: Prevention of nosocomial infection in critically ill patients by selective decontamination of the digestive tract. A randomized, double blind, placebo-controlled study. *Intensive Care Med*. 1992;18:398-404

S273. Rodríguez-Roldán JM, Altuna-Cuesta A, López A, Carrillo A, Garcia J, León J, Martínez-Pellús AJ: Prevention of nosocomial lung infection in ventilated patients: use of an antimicrobial pharyngeal nonabsorbable paste. *Crit Care Med*. 1990;18:1239-42

S274. Rolando N, Gimson A, Wade J, Philpott-Howard J, Casewell M, Williams R: Prospective controlled trial of selective parenteral and enteral antimicrobial regimen in fulminant liver failure. *Hepatol*. 1993;17:196-201

S275. Sanchez-Garcia M, Cambronero JA, Lopez-Diaz J, et al. Effectiveness and cost of selective decontamination of the digestive tract in critically ill intubated patients. A randomized, double-blind, placebo-controlled multicenter trial. *Am Rev Respir Dis* 1998; 158:908-16.

S276. Stoutenbeek CP, van Saene HKF, Little RA, Whitehead A: The effect of selective decontamination of the digestive tract on mortality in multiple trauma patients: a multicenter randomized controlled trial. *Intensive Care Med*. 2007;33:261-270

S277. Ulrich C, Harinck-deWeerd JE, Bakker NC, et al. Selective decontamination of the digestive tract with norfloxacin in the prevention of ICU-acquired infections: A prospective randomized study. *Intensive Care Med* 1989; 15: 424-31.

S278. Unertl K, Ruckdeschel G, Selbmann HK, et al; Prevention of colonization and respiratory infections in long-term ventilated patients by local antimicrobial prophylaxis. *Intensive Care Med* 1987;13:106-113

S279. Verwaest C, Verhaegen J, Ferdinande P, Schetz M, Van den Berghe G, Verbist L, Lauwers P: Randomized, controlled trial of selective digestive decontamination in 600 mechanically ventilated patients in a multidisciplinary intensive care unit. *Crit Care Med*. 1997;25:63-71

S280. Wiener J, Itokazu G, Nathan C, Kabins SA, Weinstein RA: A randomized, double-blind, placebo-controlled trial of selective digestive decontamination in a medical-surgical intensive care unit. *Clin Infect Dis*. 1995;20:861-867

S281. Huang SS, Septimus EJ, Kleinman K, Heim LT, Moody JA, Avery TR, McLean L, Rashid S, Haffenreffer K, et al. Nasal Iodophor Antiseptic vs Nasal Mupirocin Antibiotic in the Setting of Chlorhexidine Bathing to Prevent Infections in Adult ICUs: A Randomized Clinical Trial. *JAMA*. 2023;330(14):1337-47.

S282. Verhaegen J: Randomized study of selective digestive decontamination on colonization and prevention of infection in mechanically ventilated patients in the ICU. 1992. Doctor in Medical Sciences – thesis, University Hospital, Leuven, Belgium.

S283. Camus C, Sebille V, Legras A, Garo B, Renault A, Le Corre P, Donnio PY, Gacouin A, Le et al. Mupirocin/chlorexidine to prevent methicillin-resistant Staphylococcus aureus infections: post hoc analysis of a placebo-controlled, randomized trial using mupirocin/chlorhexidine and polymyxin/tobramycin for the prevention of acquired infections in intubated patients. Infection. 2014;42(3):493-502.

S284. Alkhawaja S, Martin C, Butler RJ, Gwadry-Sridhar F. Post-pyloric versus gastric tube feeding for preventing pneumonia and improving nutritional outcomes in critically ill adults. Cochrane Database of Systematic Reviews. 2015(8).

S285. Bo L, Li J, Tao T, Bai Y, Ye X, Hotchkiss RS, Kollef MH, Crooks NH, Deng X. Probiotics for preventing ventilator-associated pneumonia. Cochrane Database of Systematic Reviews 2014, Issue 10. Art. No.: CD009066.

S286. Gillies D, Todd DA, Foster JP, Batuwitage BT. Heat and moisture exchangers versus heated humidifiers for mechanically ventilated adults and children. Cochrane Database of Systematic Reviews 2017, Issue 9. Art. No.: CD004711.

S287. Solà I, Benito S. Closed tracheal suction systems versus open tracheal suction systems for mechanically ventilated adult patients. Cochrane Database of Systematic Reviews 2007, Issue 4. Art. No.: CD004581.

S288. Tokmaji G, Vermeulen H, Müller MCA, Kwakman PHS, Schultz MJ, Zaat SAJ. Silver-coated endotracheal tubes for prevention of ventilator-associated pneumonia in critically ill patients. Cochrane Database of Systematic Reviews 2015, Issue 8. Art. No.: CD009201

S289. Toews I, George AT, Peter JV, Kirubakaran R, Fontes LES, Ezekiel JPB, Meerpohl JJ. Interventions for preventing upper gastrointestinal bleeding in people admitted to intensive care units. Cochrane Database of Systematic Reviews 2018, Issue 6. Art. No.: CD008687.

S290. Wang L, Li X, Yang Z, Tang X, Yuan Q, Deng L, Sun X. Semi-recumbent position versus supine position for the prevention of ventilator-associated pneumonia in adults requiring mechanical ventilation. Cochrane Database of Systematic Reviews 2016, Issue 1. Art. No.: CD009946.

S291. Hua F, Xie H, Worthington HV, Furness S, Zhang Q, Li C. Oral hygiene care for critically ill patients to prevent ventilator-associated pneumonia. Cochrane Database of Systematic Reviews 2016, Issue 10. Art. No.: CD008367.

S292. Zhao T, Wu X, Zhang Q, Li C, Worthington HV, Hua F. Oral hygiene care for critically ill patients to prevent ventilator-associated pneumonia. Cochrane Database of Systematic Reviews 2020, Issue 12. Art. No.: CD008367.

S293. Liberati A, D'Amico R, Pifferi S, Torri V, Brazzi L, Parmelli E. Antibiotic prophylaxis to reduce respiratory tract infections and mortality in adults receiving intensive care. Cochrane Database of Systematic Reviews 2009, Issue 4. Art. No.: CD000022.

S294. Minozzi S, Pieri S, Brazzi L, Pecoraro V, Montrucchio G, D'Amico R. Topical antibiotic prophylaxis to reduce respiratory tract infections and mortality in adults receiving mechanical ventilation. Cochrane Database of Systematic Reviews 2021, Issue 1. Art. No.: CD000022.

Figure S1.
S aureus VAP prevention effect sizes;
Non decontamination interventions

Study	Treatment Yes	Treatment No	Control Yes	Control No	Odds ratio with 95% CI	Weight (%)
Miano	0	457	3	374	0.12 [0.01, 2.27]	0.29
Driks	0	61	4	65	0.12 [0.01, 2.24]	0.29
Drakulovic	0	39	4	43	0.12 [0.01, 2.35]	0.29
Kostadima	0	20	3	18	0.13 [0.01, 2.67]	0.28
Rumbak	1	59	5	55	0.19 [0.02, 1.65]	0.54
Lorente'14	1	133	5	145	0.22 [0.03, 1.89]	0.55
Dreyfuss	0	61	2	68	0.22 [0.01, 4.73]	0.27
Lacherade '10	2	167	8	156	0.23 [0.05, 1.12]	1.04
Lorente'07	2	138	8	132	0.24 [0.05, 1.15]	1.04
Mahmoodpoor	1	137	4	134	0.24 [0.03, 2.22]	0.53
Damas	1	167	3	152	0.30 [0.03, 2.95]	0.49
Holzapfel_I_99	7	192	21	179	0.31 [0.13, 0.75]	3.30
Smulders	1	74	3	72	0.32 [0.03, 3.19]	0.49
Martin	0	65	1	65	0.33 [0.01, 8.33]	0.25
Lorente'06b	2	51	5	46	0.36 [0.07, 1.95]	0.90
Pickworth	0	39	1	43	0.37 [0.01, 9.27]	0.24
Prod'hom_S	2	81	5	76	0.38 [0.07, 1.99]	0.92
Walaszek	2	1,001	4	800	0.40 [0.07, 2.19]	0.88
Manzano	4	60	9	54	0.40 [0.12, 1.37]	1.68
Pneumatikos	2	37	4	36	0.49 [0.08, 2.82]	0.83
Morrow	8	65	14	59	0.52 [0.20, 1.32]	2.91
Kollef'08_silverETT	9	757	16	727	0.54 [0.24, 1.23]	3.77
Reigneir	10	217	17	205	0.56 [0.25, 1.24]	3.95
Dreyfuss	1	27	2	33	0.61 [0.05, 7.11]	0.42
Zeng	12	106	16	101	0.71 [0.32, 1.58]	4.02
Marjanović_AGATE	21	195	27	191	0.76 [0.42, 1.39]	6.99
Cook	36	568	44	552	0.80 [0.50, 1.25]	12.29
Kollef '97_post	4	323	5	322	0.80 [0.21, 3.00]	1.46
Francois_Saatellite	43	153	26	74	0.80 [0.46, 1.40]	8.13
Prod'hom_R	4	76	5	76	0.80 [0.21, 3.09]	1.40
Thomachot	5	66	7	77	0.83 [0.25, 2.75]	1.79
Lorente '04	14	147	14	129	0.88 [0.40, 1.91]	4.22
Lorente '03	7	107	8	108	0.88 [0.31, 2.52]	2.32
Lorente'06a	8	228	8	213	0.93 [0.34, 2.53]	2.57
Valencia	2	71	2	67	0.94 [0.13, 6.89]	0.65
Knight	0	130	0	129	0.99 [0.02, 50.39]	0.17
Kirton	6	134	6	134	1.00 [0.31, 3.18]	1.91
Nseir	1	60	1	60	1.00 [0.06, 16.36]	0.33
Staudinger	2	73	2	73	1.00 [0.14, 7.29]	0.65
Thomachot	7	56	8	69	1.08 [0.37, 3.16]	2.21
Thomachot	8	62	7	59	1.09 [0.37, 3.19]	2.21
Lorente'05	10	200	10	223	1.11 [0.45, 2.73]	3.17
Lacherade '05	18	167	16	168	1.13 [0.56, 2.29]	5.11
Forestier	12	90	11	95	1.15 [0.48, 2.74]	3.39
Dat	7	289	6	295	1.19 [0.40, 3.59]	2.10
Bonten '95	7	67	4	63	1.65 [0.46, 5.89]	1.57
Ryan_S	2	56	1	55	1.96 [0.17, 22.29]	0.43
Acosta-escribano	8	42	4	50	2.38 [0.67, 8.46]	1.59
Heyland	1	48	0	46	2.88 [0.11, 72.41]	0.25
Laueny	17	81	6	85	2.97 [1.12, 7.92]	2.66
Djedaini	2	66	0	61	4.62 [0.22, 98.24]	0.27
**Overall**					0.76 [0.65, 0.90]	

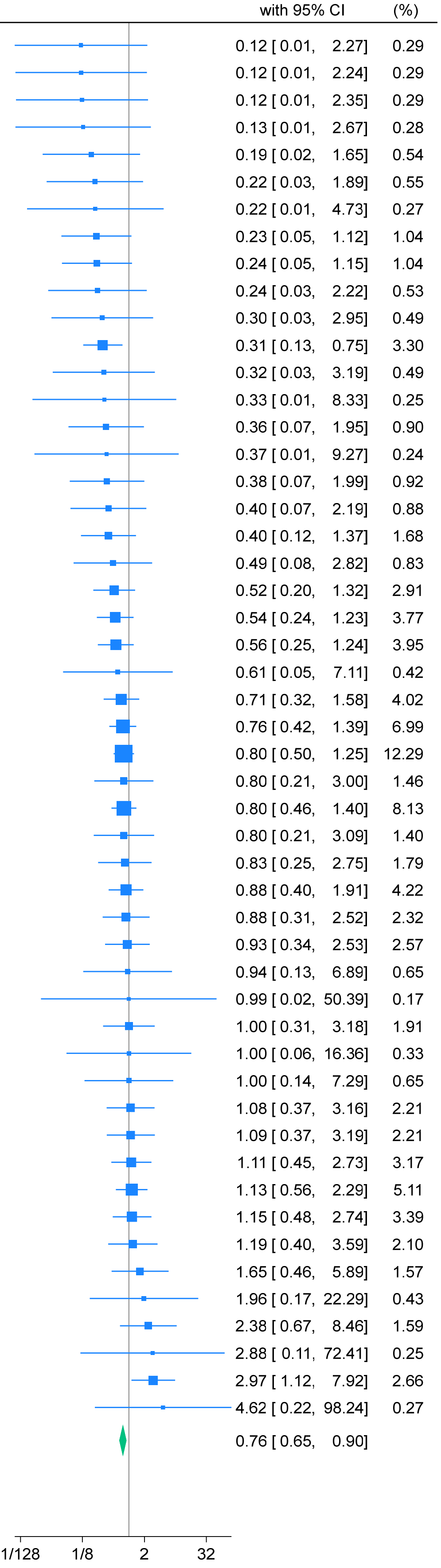

Heterogeneity: $\tau^2 = 0.00$, $I^2 = 0.00\%$, $H^2 = 1.00$

Test of $\theta_i = \theta_j$: $Q(50) = 45.13$, $p = 0.67$

Test of $\theta = 0$: $z = -3.32$, $p = 0.00$

Random-effects REML model
Sorted by: _meta_es

Figure S2.
S aureus VAP prevention effect sizes; Decontamination interventions

Study	Treatment Yes	Treatment No	Control Yes	Control No	Odds ratio with 95% CI	Weight (%)
**Antiseptic intervention**						
Fourrier'00	0	30	3	27	0.13 [0.01, 2.61]	0.36
Segers	1	484	6	463	0.16 [0.02, 1.33]	0.72
Cabov	0	30	2	28	0.19 [0.01, 4.06]	0.34
Caruso	1	129	5	127	0.20 [0.02, 1.71]	0.69
Morris	7	494	75	1,385	0.26 [0.12, 0.57]	4.62
Seguin-PVI	3	33	14	48	0.31 [0.08, 1.17]	1.78
Mori	7	1,241	7	407	0.33 [0.11, 0.94]	2.72
Camus '05 MCh	3	127	8	118	0.35 [0.09, 1.34]	1.71
Koeman-Ch	2	125	5	125	0.40 [0.08, 2.10]	1.16
Fourrier'05	1	113	2	112	0.50 [0.04, 5.54]	0.56
Kollef'06	17	345	25	322	0.63 [0.34, 1.20]	6.52
Seguin-PVI	14	64	14	58	0.91 [0.40, 2.06]	4.24
Lorente'12	4	213	4	215	1.01 [0.25, 4.09]	1.60
Heterogeneity: τ^2 = 0.07, I^2 = 14.48%, H^2 = 1.17					0.44 [0.30, 0.64]	
Test of $\theta_i = \theta_j$: Q(12) = 10.31, p = 0.59						
Test of θ = 0: z = -4.27, p = 0.00						
**Antibiotic intervention**						
Camus '14	0	1,022	17	908	0.03 [0.00, 0.42]	0.41
Winter NC	0	91	4	80	0.10 [0.01, 1.84]	0.38
Stoutenbeek'87 SDD	3	60	18	41	0.11 [0.03, 0.41]	1.88
Camus '05 PT&MCh	1	128	8	118	0.12 [0.01, 0.94]	0.74
Blair	1	125	7	123	0.14 [0.02, 1.16]	0.72
Unertl	1	18	5	15	0.17 [0.02, 1.59]	0.64
Aerdts	0	17	4	35	0.23 [0.01, 4.43]	0.37
Wiener	1	29	4	27	0.23 [0.02, 2.22]	0.64
Langlois-Karaga	5	42	15	35	0.28 [0.09, 0.84]	2.48
Rocha	5	42	15	39	0.31 [0.10, 0.93]	2.50
Winter	0	91	1	91	0.33 [0.01, 8.29]	0.32
Georges	0	31	1	32	0.34 [0.01, 8.76]	0.31
Hartenauer	3	96	8	93	0.36 [0.09, 1.41]	1.70
Stoutenbeek '07	18	183	40	160	0.39 [0.22, 0.71]	7.21
Cerra	5	20	8	13	0.41 [0.11, 1.52]	1.79
Rodríguez-Roldán	0	13	1	16	0.41 [0.02, 10.83]	0.30
Ulrich	2	46	5	47	0.41 [0.08, 2.21]	1.12
Korinek	9	54	16	44	0.46 [0.18, 1.14]	3.55
Bonten NC	0	22	2	52	0.47 [0.02, 10.12]	0.34
Quinio	9	67	16	56	0.47 [0.19, 1.15]	3.68
Palomar_1	5	36	8	34	0.59 [0.18, 1.98]	2.10
Stoutenbeek'87 ED	9	33	18	41	0.62 [0.25, 1.56]	3.46
Verwaest OA	6	187	9	176	0.63 [0.22, 1.80]	2.72
de la Cal	12	41	16	38	0.70 [0.29, 1.66]	3.84
Camus '05 PT	6	124	8	118	0.71 [0.24, 2.12]	2.56
Sanchez-Garcia	5	126	7	133	0.75 [0.23, 2.44]	2.23
Biagioni_COVID	7	79	24	238	0.88 [0.36, 2.12]	3.76
Bergmans	3	84	3	75	0.89 [0.17, 4.56]	1.19
Abele-Horn	9	49	5	25	0.92 [0.28, 3.03]	2.16
Verwaest PTA	9	191	9	176	0.92 [0.36, 2.37]	3.30
KoemanChC	5	123	5	125	1.02 [0.29, 3.60]	1.94
Laggner	0	33	0	34	1.03 [0.02, 53.41]	0.21
Nardi '90	3	44	3	47	1.07 [0.20, 5.57]	1.16
Rouby	21	326	14	237	1.09 [0.54, 2.19]	5.61
Hammond	4	35	3	30	1.14 [0.24, 5.52]	1.28
Jacobs	0	36	0	43	1.19 [0.02, 61.56]	0.21
Karvouniaris	5	79	4	80	1.27 [0.33, 4.89]	1.71
Ferrer	3	36	2	39	1.62 [0.26, 10.29]	0.94
Massart_ECMO	2	67	3	169	1.68 [0.27, 10.29]	0.97
Rolando '93	2	26	1	30	2.31 [0.20, 26.94]	0.54
Heterogeneity: τ^2 = 0.02, I^2 = 4.39%, H^2 = 1.05					0.57 [0.46, 0.70]	
Test of $\theta_i = \theta_j$: Q(39) = 38.27, p = 0.50						
Test of θ = 0: z = -5.29, p = 0.00						
Heterogeneity: τ^2 = 0.03, I^2 = 6.09%, H^2 = 1.06						
Test of $\theta_i = \theta_j$: Q(52) = 49.85, p = 0.56						
Test of θ = 0: z = -6.81, p = 0.00						
Test of group differences: $Q_b(1)$ = 1.34, p = 0.25						

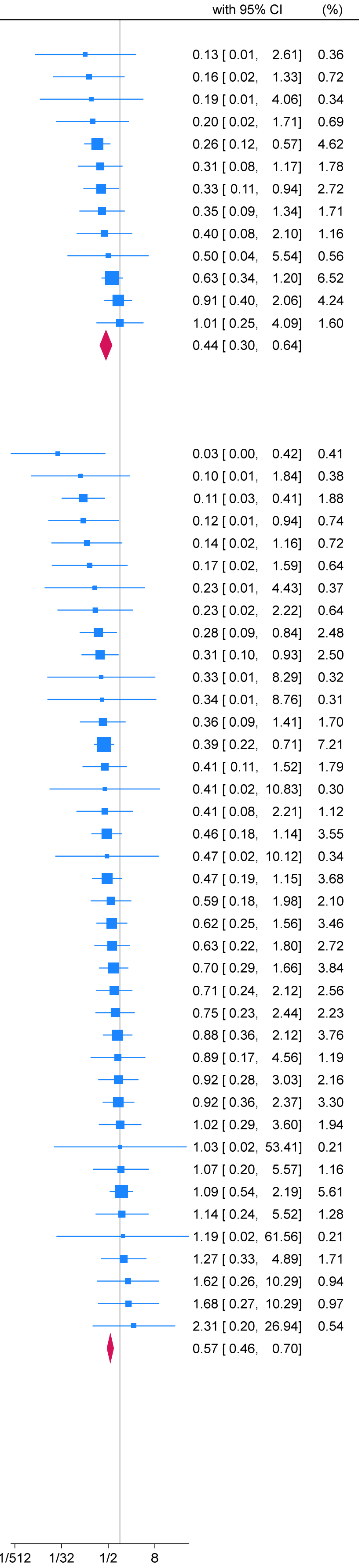

Random-effects REML model
Sorted by: _meta_es

Figure S3.
S aureus BSI prevention effect sizes;
All interventions

Study	Treatment Yes	Treatment No	Control Yes	Control No	Odds ratio with 95% CI	Weight (%)
**Non-decontamination intervention**						
Kollef '97_post	1	326	5	348	0.21 [0.02, 1.84]	0.95
Montecalvo_G	1	18	2	17	0.47 [0.04, 5.70]	0.72
Francois_Saatellite	10	186	7	93	0.71 [0.26, 1.94]	3.48
Holzapfel_I_99	2	197	2	198	1.01 [0.14, 7.21]	1.11
Laggner	1	15	0	16	2.13 [0.07, 68.36]	0.38
Heterogeneity: $\tau^2 = 0.00$, $I^2 = 0.00\%$, $H^2 = 1.00$					0.66 [0.31, 1.40]	
Test of $\theta_i = \theta_j$: Q(4) = 1.76, p = 0.78						
Test of $\theta = 0$: z = -1.09, p = 0.28						
**Antiseptic intervention**						
Cabov	0	30	2	28	0.19 [0.01, 4.06]	0.48
Fourrier'00	0	30	1	29	0.32 [0.01, 8.24]	0.44
Bleasdale	0	391	1	444	0.38 [0.02, 9.32]	0.45
Huang_3UD	92	25,932	80	17,276	0.77 [0.57, 1.03]	10.37
Milstone	3	2,419	4	2,521	0.78 [0.17, 3.50]	1.81
Huang_2TD	106	24,646	70	15,148	0.93 [0.69, 1.26]	10.33
Climo_CRT	9	1,401	8	1,390	1.12 [0.43, 2.90]	3.70
Noto_post	16	4,472	15	4,837	1.15 [0.57, 2.34]	5.48
Fourrier'05	1	113	0	114	2.02 [0.07, 60.74]	0.40
Wittekamp Chlx	25	2,083	13	2,238	2.07 [1.05, 4.05]	5.78
Heterogeneity: $\tau^2 = 0.04$, $I^2 = 26.51\%$, $H^2 = 1.36$					0.98 [0.74, 1.30]	
Test of $\theta_i = \theta_j$: Q(9) = 9.51, p = 0.39						
Test of $\theta = 0$: z = -0.13, p = 0.90						
**Antibiotic intervention**						
Stoutenbeek'87 SDD	0	63	7	52	0.06 [0.00, 0.99]	0.54
Ferrer	0	39	2	39	0.20 [0.01, 4.30]	0.48
Beovic	0	81	2	95	0.23 [0.01, 4.95]	0.49
de Smet NC SDD	9	2,036	22	1,968	0.40 [0.18, 0.86]	4.87
Rimola	2	66	5	67	0.41 [0.08, 2.17]	1.49
de Smet NC SOD	9	1,895	22	1,968	0.42 [0.20, 0.93]	4.87
Gaussorgues	1	58	2	57	0.49 [0.04, 5.57]	0.76
Rolando '93	0	28	0	31	0.55 [0.02, 17.10]	0.39
Stoutenbeek'87 ED	3	39	7	52	0.57 [0.14, 2.35]	2.00
Jacobs	0	36	0	43	0.60 [0.02, 18.27]	0.39
Camus '14	2	1,020	3	922	0.60 [0.10, 3.61]	1.32
SuDDICU	27	2,764	46	3,145	0.67 [0.41, 1.08]	7.96
Rocha	2	45	3	51	0.76 [0.12, 4.73]	1.27
Hartenauer	3	96	4	97	0.76 [0.17, 3.48]	1.76
Massart_ECMO	0	69	1	171	0.82 [0.03, 20.44]	0.44
Wittekamp PTNy	12	2,212	13	2,238	0.93 [0.43, 2.05]	4.80
Wittekamp PTNy	17	2,065	13	2,238	1.42 [0.69, 2.93]	5.31
Stoutenbeek '07	8	193	5	195	1.62 [0.52, 5.03]	2.86
Camus '05 PT&MCh	2	127	1	125	1.97 [0.18, 21.99]	0.76
Quinio	8	68	4	68	2.00 [0.58, 6.96]	2.46
Laggner	1	32	0	34	2.12 [0.07, 65.54]	0.39
Verwaest OA	7	186	3	182	2.28 [0.58, 8.97]	2.12
Camus '05 PT	3	127	1	125	2.95 [0.30, 28.77]	0.85
de la Cal	14	39	6	53	3.17 [1.12, 8.99]	3.26
Verwaest PTA	10	190	3	182	3.19 [0.86, 11.79]	2.28
Heterogeneity: $\tau^2 = 0.22$, $I^2 = 35.48\%$, $H^2 = 1.55$					0.92 [0.65, 1.29]	
Test of $\theta_i = \theta_j$: Q(24) = 33.23, p = 0.10						
Test of $\theta = 0$: z = -0.50, p = 0.62						
Heterogeneity: $\tau^2 = 0.10$, $I^2 = 28.11\%$, $H^2 = 1.39$						
Test of $\theta_i = \theta_j$: Q(39) = 45.37, p = 0.22						
Test of $\theta = 0$: z = -0.76, p = 0.45						
Test of group differences: $Q_b(2) = 0.96$, p = 0.62						

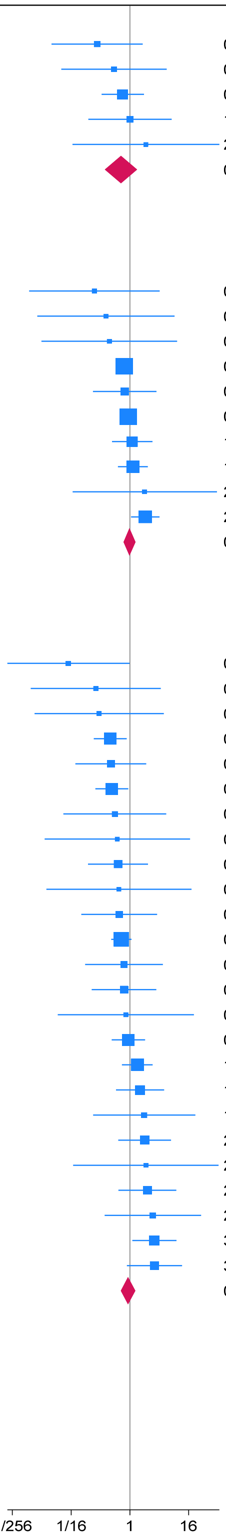

Random-effects REML model
Sorted by: _meta_es

Figure S4. Meta-regression of S aureus VAP prevention effect sizes versus LOS, studies of Non-decontamination interventions labelled with reference numbers

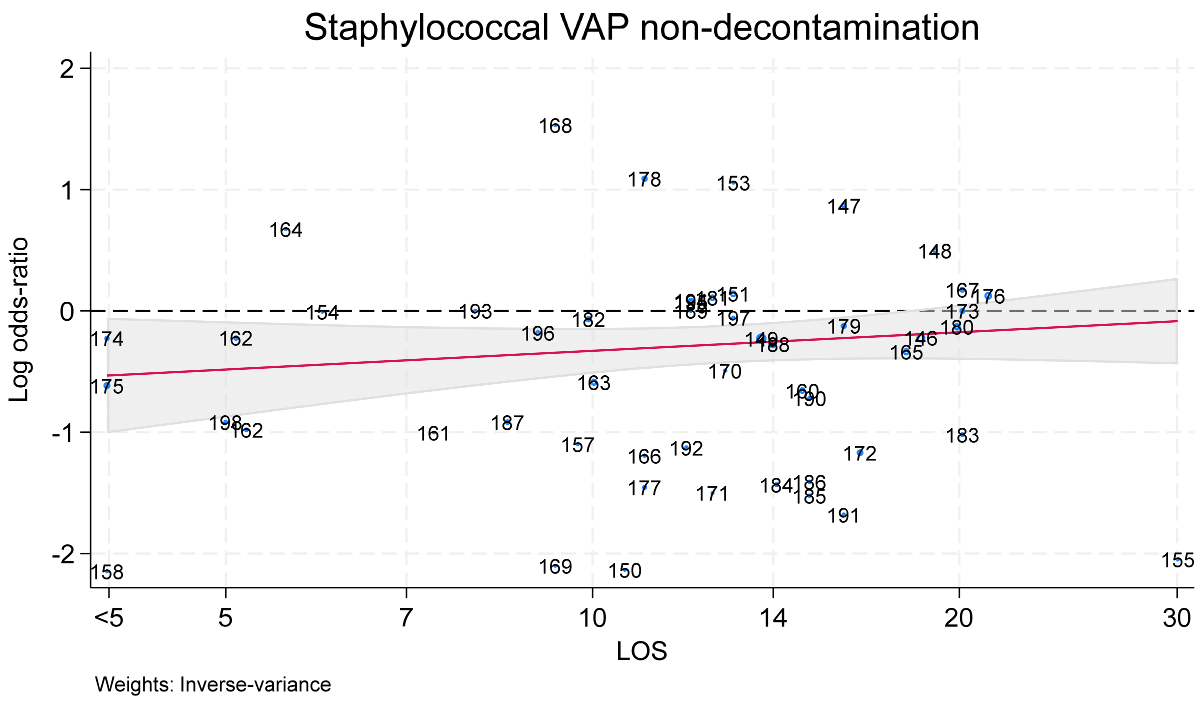

Figure S5. Meta-regression of S aureus BSI prevention effect sizes versus LOS, studies of Non-decontamination interventions labelled with reference numbers

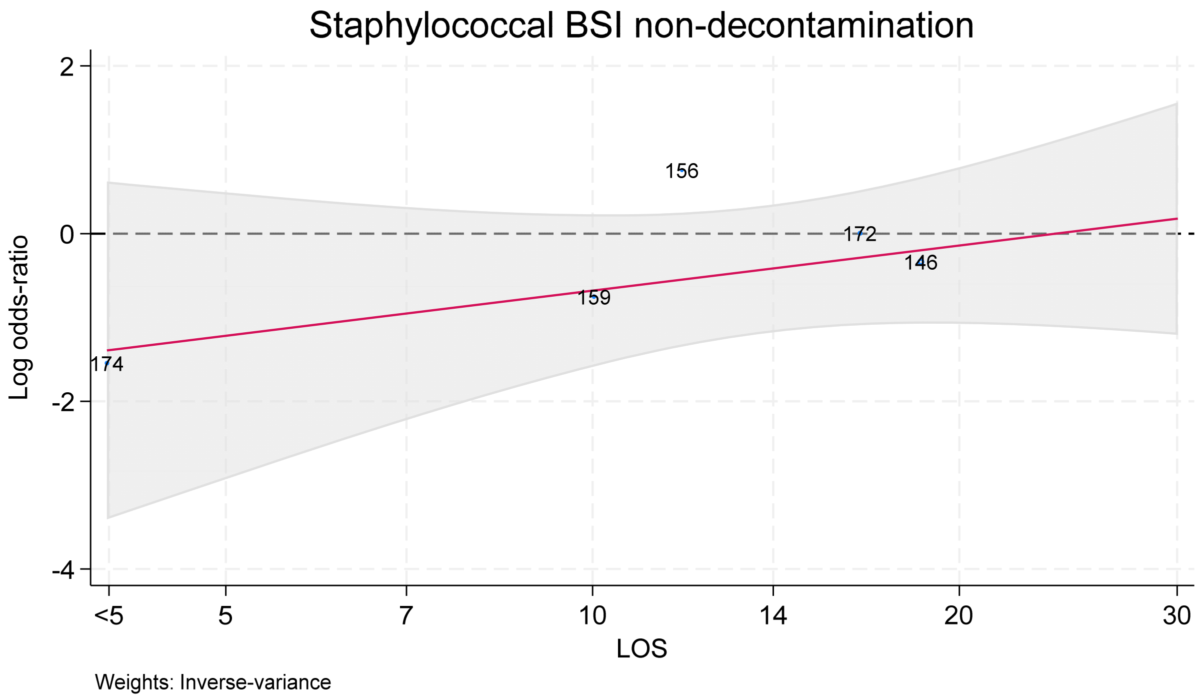

Figure S6. Meta-regression of S aureus VAP prevention effect sizes versus LOS, studies of Anti-septic interventions labelled with reference numbers

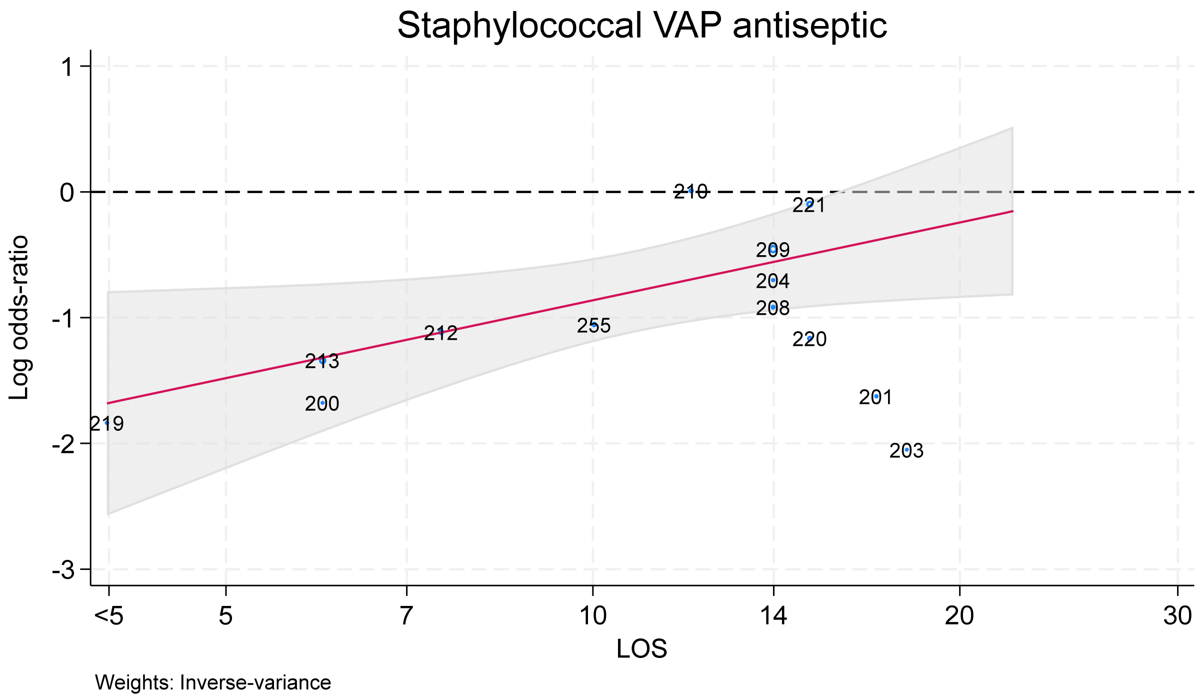

Figure S7. Meta-regression of S aureus BSI prevention effect sizes versus LOS, studies of Anti-septic interventions labelled with reference numbers

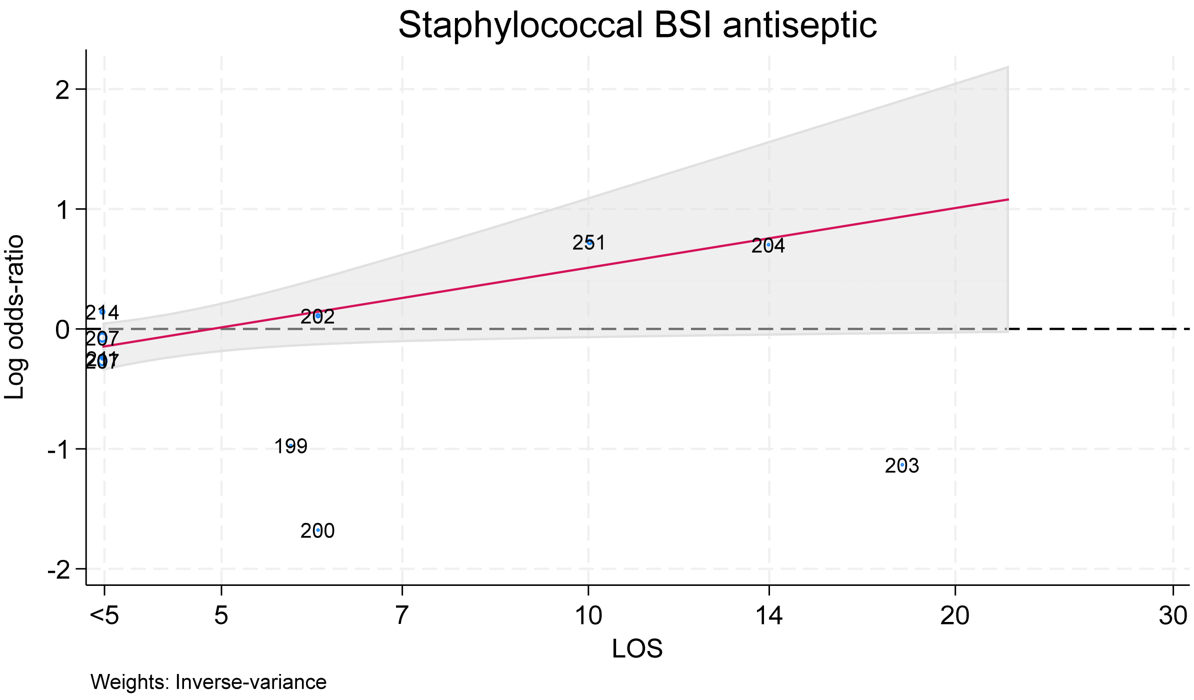

Figure S8. Meta-regression of S aureus VAP prevention effect sizes versus LOS, studies of Antibiotic interventions labelled with reference numbers

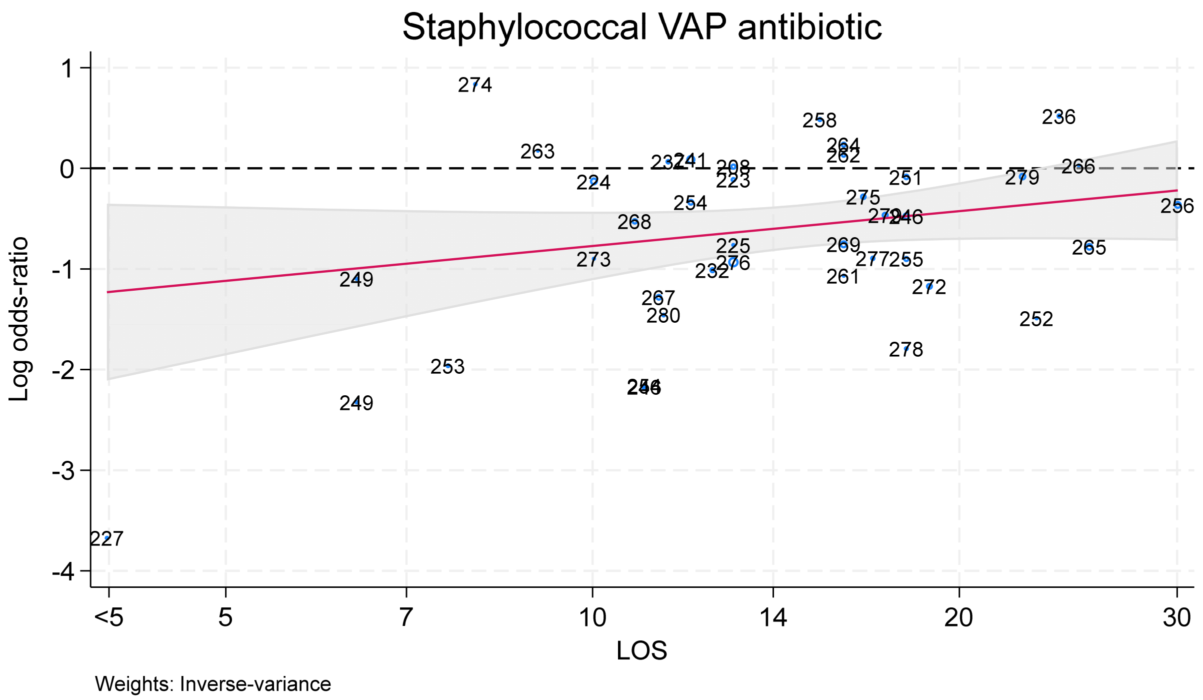

Figure S9. Meta-regression of S aureus BSI prevention effect sizes versus LOS, studies of Antibiotic interventions labelled with reference numbers

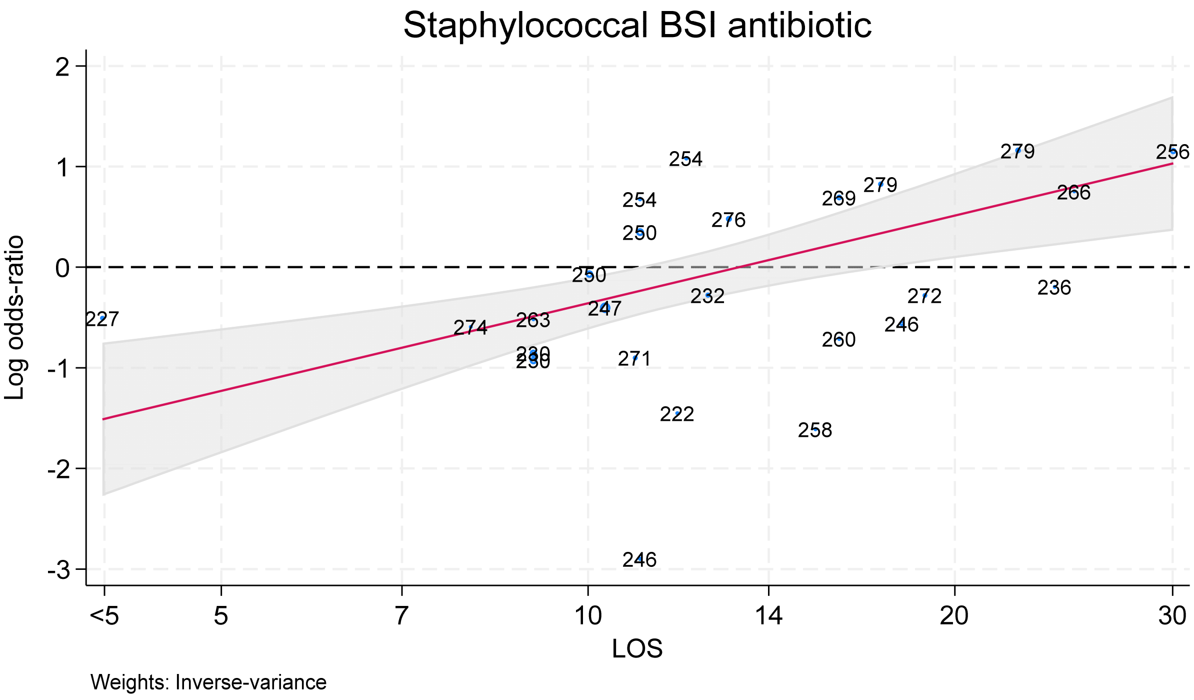

Figure S10. Meta-regression of S aureus VAP incidence versus LOS among observational cohorts and control groups of non-concurrent control studies labelled with reference numbers. These regression lines serve as benchmark in the meta-regression models presented in Table S6

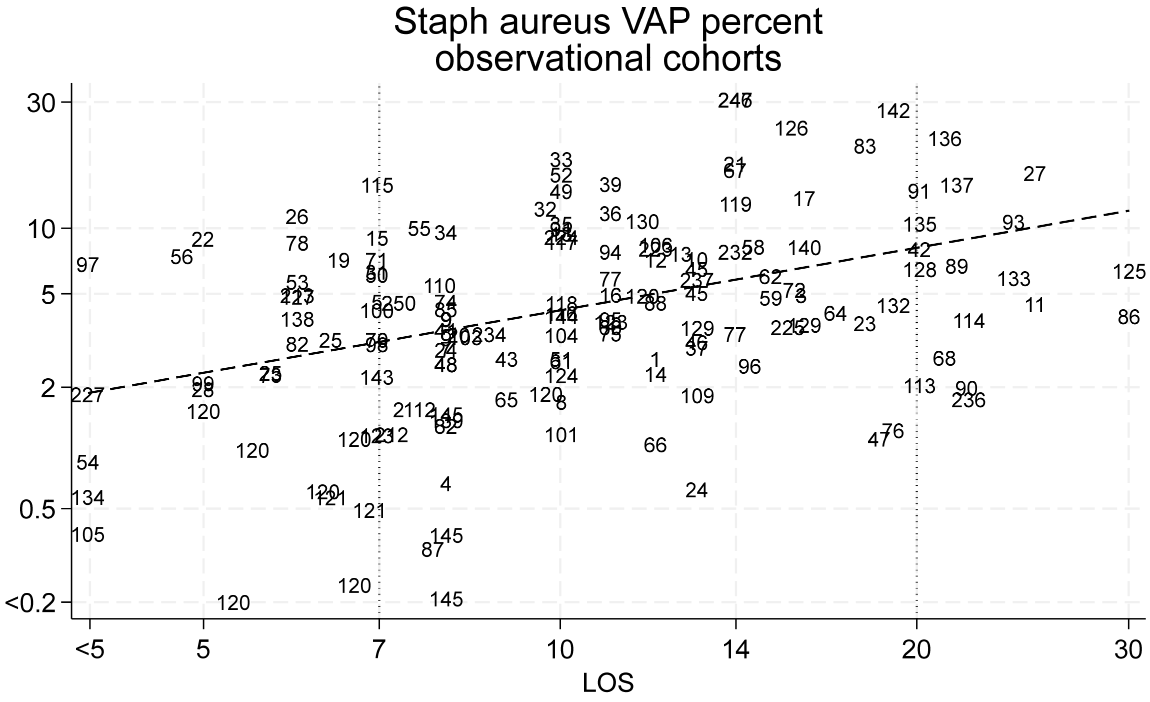

Figure S11. Meta-regression of S aureus BSI incidence versus LOS among observational cohorts and control groups of non-concurrent control studies labelled with reference numbers. These regression lines serve as benchmark in the meta-regression models presented in Table S6

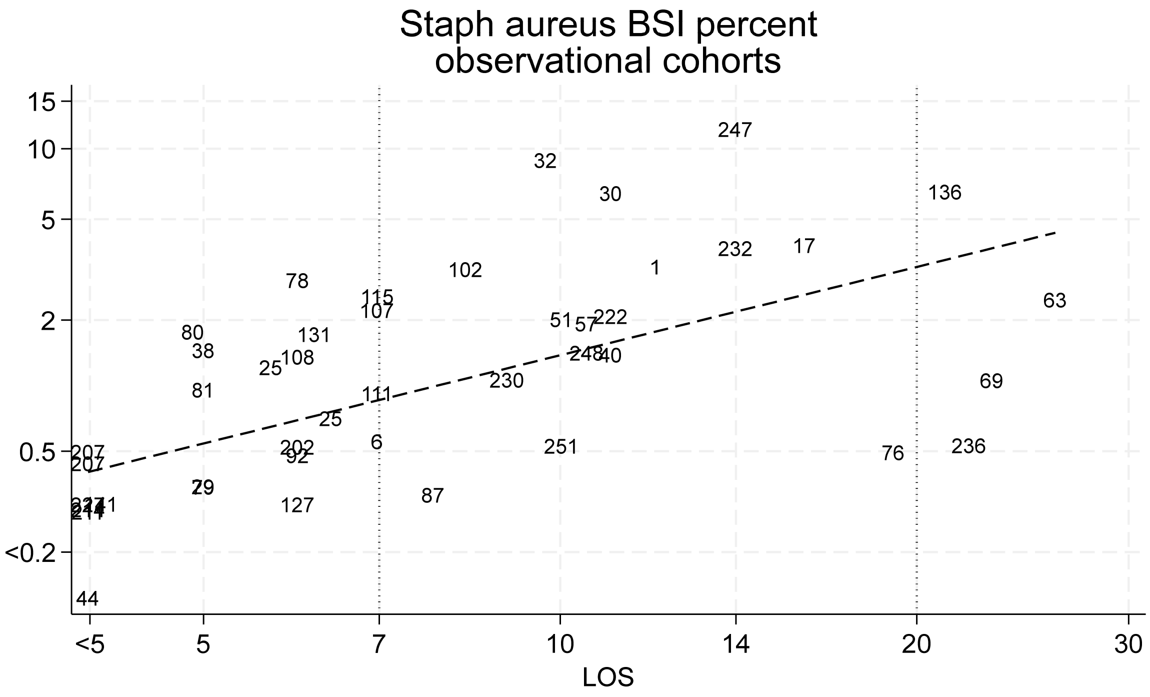

Figure S12. GSEM of postulated model of causation as presented in Figure 3 but not including the interaction terms.

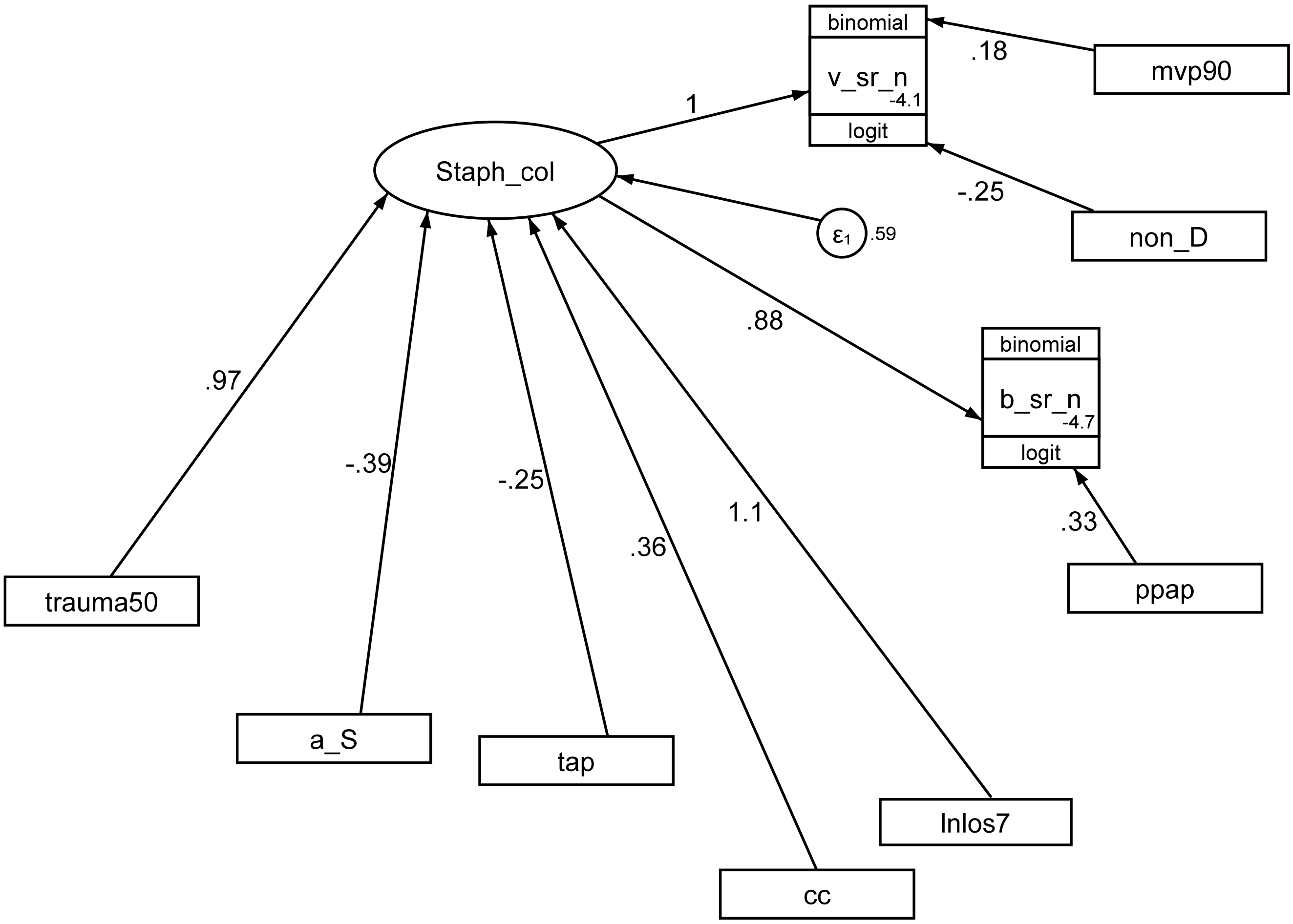

Supplement: Supplementary file 1 [file antibiotics-13-00316-s001.zip › antibiotics-2910098-supplementary.pdf]
